# Cathagines A–D, new bisindole alkaloids from *Catharanthus roseus*

**DOI:** 10.1007/s11418-024-01857-4

**Published:** 2024-11-14

**Authors:** Yusuke Hirasawa, Chiaki Kasagi, Erika Koyama, Hitomi Myojin, Takahiro Tougan, Toshihiro Horii, Nahoko Uchiyama, Toshio Kaneda, Hiroshi Morita

**Affiliations:** 1 Faculty of Pharmaceutical Sciences, Hoshi University, Ebara 2-4-41 Shinagawa-ku, Tokyo, 142-8501 Japan; 2 Research Center for Infectious Disease Control, Research Institute for Microbial Diseases, Osaka University, 3-1 Yamadaoka, Suita, Osaka 565-0871 Japan; 3 Department of Malaria Vaccine Development, Research Institute for Microbial Diseases, Osaka University, 3-1 Yamadaoka, Suita, Osaka 565-0871 Japan; 4 National Institute of Health Sciences, 3-25-26 Tonomachi, Kawasaki-ku, Kawasaki, Kanagawa 210-9501 Japan

**Keywords:** Bisindole alkaloid, *Catharanthus roseus*, Apocynaceae, Cathagines A–D, Aspidosperma, 3-spirooxindole, Antiplasmodial activity

## Abstract

Four new bisindole alkaloids, cathagines A (**1**)–D (**4**) consisting of an aspidosperma and the fused tetracyclic 3-spirooxindole derived from an iboga type skeleton were isolated from the whole plant of *Catharanthus roseus*. The structures including absolute stereochemistry were elucidated on the basis of 2D NMR data and CD spectra. Cathagine B (**2**) showed moderate anti-malarial activity against *Plasmodium falciparum* 3D7.

**Graphical abstract:**

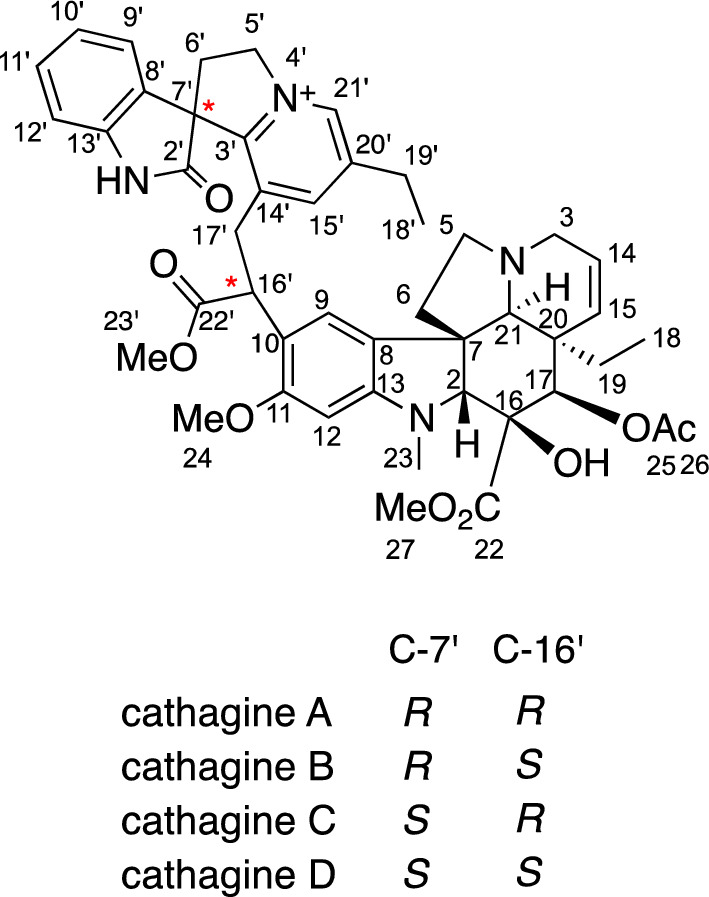

**Supplementary Information:**

The online version contains supplementary material available at 10.1007/s11418-024-01857-4.

## Introduction

Plants belonging to the Apocynaceae family are known to contain various kinds of monoterpene indole alkaloids [1]. Among them, *Catharanthus roseus* (L.) G. Don, which is native to Madagascar, is well known for producing a various kind of indole alkaloids [2]. Especially, studies on anticancer dimeric indole alkaloids such as vinblastine and vincristine have been conducted for many years [3].

**Fig. 1:**
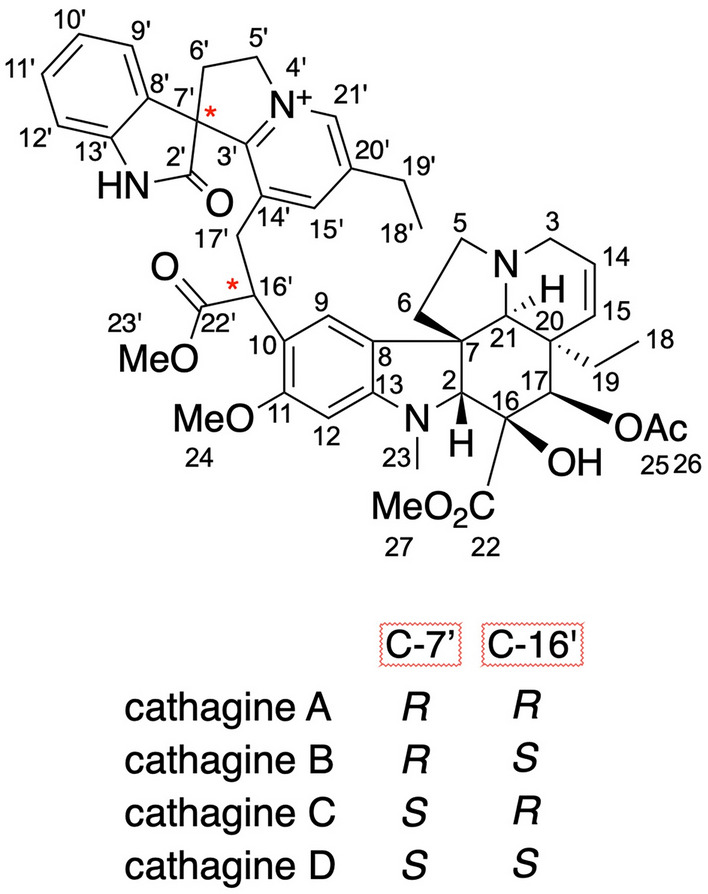
Structures of cathagines A–D (**1**–**4**)

In the search for new bioactive alkaloids from tropical plants such as *Hunteria zeylanica* [4–7], *Leuconotis griffithii* [8–12], *Tabernaemontana macrocarpa* [13], and *Tabernaemontana divaricata* [14, 15], a preliminary study of the plant of *Catharanthus roseus* with cytotoxic activity against leukemia cell line and antimalarial activity resulted in the isolation of new bisindole alkaloids, dimeric isovincathicine [16], dimeric vincazalidine A [17], and trimeric vincarostine A [18]. Further investigation on the extract of *C. roseus* yielded four new bisindole alkaloids, cathagines A–D (**1**–**4**) consisting of an aspidosperma and the fused tetracyclic 3-spirooxindole (Fig. 1), and their structure elucidation on the basis of their spectral data and antimalarial activity are reported herein.

## Results and discussion

### Structure elucidation of cathagines A (1)–D (4)

Cathagines A-D were obtained as yellow solid, and the ESIMS displayed a molecular ion peak at 805 (M)^+^ and each molecular formula was estimated to be C_46_H_53_N_4_O_9_ from HR-ESI–MS. IR absorptions indicated the presence of hydroxyl (ca. 3450 cm^–1^), an ester carbonyl group (ca. 1730 cm^–1^), and a lactam group (ca. 1690 cm^–1^) in each case. The specific rotations were cathagine A {[α] _D_^23^ – 133 (*c* 1.0, MeOH)}, cathagine B {[α] _D_^23^ + 75 (*c* 1.0, MeOH)}, cathagine C {[α] _D_^23^ + 95 (*c* 1.0, MeOH)}, cathagine D {[α] _D_^23^ – 143 (*c* 1.0, MeOH)}. Based on the ^1^H NMR spectra of them, each of cathagines A-D is suggested to be a stereoisomer. Analysis of the ^1^H and ^13^C NMR data (Tables 1, 2) and the HSQC spectrum revealed the presence of four sp^3^ quaternary carbons, four sp^3^ methines, eight sp^3^ methylenes, seven methyls, ten sp^2^ methines, and thirteen sp^2^ quaternary carbons.

**Table 1:** ^1^H (^a^600 MHz or ^b^400 MHz) NMR data of cathagines A–D (**1**–**4**) in CD_3_OD

No	**1**^a^	**2**^a^	**3**^a^	**4**^b^
2	3.58 (1H, s)	3.59 (1H, s)	3.55 (1H, s)	3.61 (1H, s)
3a	2.88 (1H, m)	2.92 (1H, brd, 16.5)	2.87 (1H, m)	2.92 (1H, m)
3b	2.91 (1H, m)	3.46 (1H, m)	3.46 (1H, dd, 16.1, 4.4)	3.50 (1H, m)
5a	2.63 (1H, m)	2.64 (1H, m)	2.59 (1H, m)	2.66 (1H, m)
5b	3.41 (1H, m)	3.35 (1H, m)	3.35 (1H, m)	3.43 (1H, m)
6a	2.24 (2H, m)	2.05 (1H, m)	1.99 (1H, m)	2.26 (1H, m)
6b		2.20 (1H, m)	2.14 (1H, m)	2.42 (1H, m)
9	6.66 (1H, s)	6.51 (1H, s)	6.50 (1H, s)	6.77 (1H, s)
12	6.14 (1H, s)	6.19 (1H, s)	6.22 (1H, s)	6.13 (1H, s)
14	5.87 (1H, dd, 9,2, 3.9)	5.88 (1H, dd, 10.0, 3.8)	5.87 (1H, dd, 9.7, 3.8)	5.87 (1H, dd, 12.0, 4.0)
15	5.16 (1H, d, 10.1)	5.18 (1H, d, 10.0)	5.16 (1H, d, 12.1)	5.15 (1H, d, 8.0)
17	5.24 (1H, s)	5.32 (1H, s)	5.32 (1H, s)	5.26 (1H, s)
18	0.34 (3H, t, 7.1)	0.44 (3H, t, 7.2)	0.44 (3H, t, 7.2)	0.36 (3H, t, 8.0)
19a	0.85 (1H, m)	1.08 (1H, m)	1.04 (1H, m)	0.90 (1H, m)
19b	1.47 (1H, m)	1.60 (1H, m)	1.58 (1H, m)	1.50 (1H, m)
21	2.66 (1H, s)	2.75 (1H, s)	2.64 (1H, s)	2.66 (1H, s)
23	2.66 (3H, s)	2.67 (3H, s)	2.64 (3H, s)	2.66 (3H, s)
24	3.67 (3H, s)	3.63 (3H, s)	3.68 (3H, s)	3.62 (3H, s)
26	1.99 (3H, s)	2.00 (3H, s)	2.00 (3H, s)	1.99 (3H, s)
27	3.76 (3H, s)	3.76 (3H, s)	3.55 (3H, s)	3.76 (3H, s)
5'a	5.06 (1H, m)	5.11 (1H, m)	5.07 (1H, m)	5.15 (2H, m)
5'b	5.16 (1H, m)	5.20 (1H, m)	5.20 (1H, m)	
6'a	2.81 (1H, m)	2.82 (1H, m)	2.86 (1H, m)	2.80 (1H, m)
6'b	2.94 (1H, m)	2.97 (1H, m)	2.98 (1H, m)	2.91 (1H, m)
9'	7.13 (1H, d, 7.2)	7.25 (1H, d, 7.3)	7.19 (1H, d, 7.2)	7.23 (1H, d, 4.0)
10'	6.96 (1H, t, 7.2)	7.00 (1H, t, 7.4)	7.03 (1H, t, 7.6)	7.00 (1H, t, 8.0)
11'	7.31 (1H, t, 7.2)	7.30 (1H, t, 7.8)	7.37 (1H, t, 7.6)	7.29 (1H, m)
12'	7.05 (1H, d, 7.5)	7.05 (1H, d, 8.0)	7.08 (1H, d, 7.6)	7.05 (1H, m)
15'	7.63 (1H, s)	7.19 (1H, s)	7.22 (1H, s)	7.30 (1H, m)
16'	4.19 (1H, dd, 10.2, 5.4)	3.22 (1H, dd, 11.3, 3.4)	3.79 (1H, m)	3.65 (1H, m)
17'a	2.72 (1H, m)	2.73 (1H, m)	2.69 (1H, m)	2.81 (1H, m)
17'b	2.90 (1H, m)	3.07 (1H, dd, 14.1, 3.8)	2.85 (1H, m)	2.95 (1H, m)
18'	1.18 (3H, t, 7.5)	1.13 (3H, t, 7.6)	1.09 (3H, t, 7.6)	1.10 (3H, t, 8.0)
19'	2.73 (2H, m)	2.68 (2H, m)	2.66 (2H, m)	2.66 (2H, m)
21'	8.77 (1H, s)	8.78 (1H, s)	8.80 (1H, s)	8.73 (1H, s)
23'	3.50 (3H, s)	3.47 (3H, s)	3.76 (3H, s)	3.43 (3H, s)

**Table 2:** ^13^C (^a^150 MHz or ^b^100 MHz) NMR data of cathagines A–D (**1**–**4**) in CD_3_OD

No	**1**^**a**^	**2**^**a**^	**3**^**a**^	**4**^**b**^
2	84.4	84.4	84.6	84.5
3	51.9	51.8	51.9	51.9
5	52.3	52.3	52.5	52.5
6	45.0	45.0	44.9	45.0
7	54.3	54.2	54.1	54.2
8	125.8	126.2	119.2	126.5
9	123.7	124.1	125.7	123.2
10	117.0	117.0	117.4	116.8
11	159.7	159.8	159.8	159.7
12	94.4	94.7	94.8	94.6
13	154.7	154.9	154.8	154.7
14	125.9	125.9	125.9	125.9
15	131.3	131.1	131.2	131.1
16	81.0	80.9	80.8	80.9
17	77.5	77.5	77.5	77.5
18	8.0	8.0	8.1	8.1
19	31.8	32.2	32.1	31.9
20	44.2	44.3	44.3	44.2
21	67.4	67.3	67.7	67.6
22	173.4	173.4	174.7	173.4
23	38.9	38.9	39	38.9
24	56.2	56.2	56.1	56.3
25	172.4	172.4	172.4	172.4
26	20.8	20.8	20.8	20.8
27	52.9	52.9	52.6	52.9
2'	181.0	181.8	180.9	N.D
3'	154.8	154.9	154.2	155.3
5'	58.6	58.9	58.6	58.8
6'	36.0	35.4	36.0	35.3
7'	62.3	62.4	62.1	62.8
8'	132.7	133.5	132.1	132.9
9'	124.4	124.7	124.7	124.8
10'	123.1	122.9	123.6	122.6
11'	131.4	131.4	131.5	131.3
12'	113.9	114.4	113.6	114.8
13'	151.0	150.8	149.2	151.2
14'	138.5	138.4	138.7	138.3
15'	147.3	147.8	148.6	147.2
16'	43.3	44.5	46.7	42.1
17'	32.9	33.4	32.6	35.4
18'	14.8	15.5	15.5	14.9
19'	26.3	26.4	26.3	26.2
20'	144.3	144.3	144.3	143.9
21'	140.1	140.2	140.4	139.9
22'	174.9	174.5	173.4	174.8
23'	52.5	52.4	52.9	52.3

**Fig. 2:**
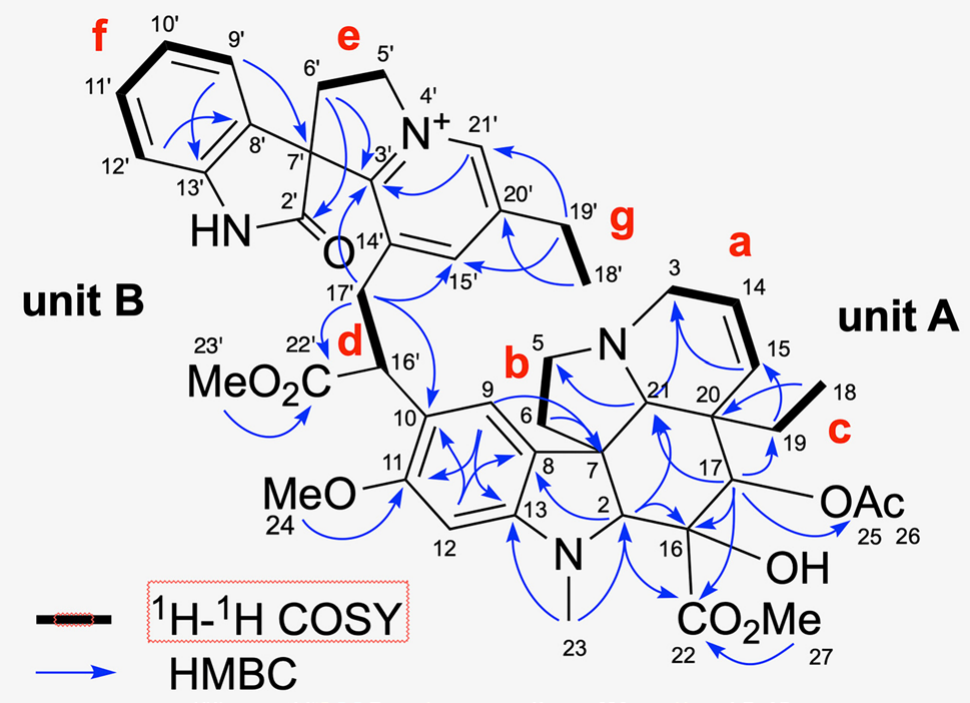
Selected 2D NMR correlations for cathagine A (**1**)

The planar structure of **1** was deduced from analyses of the 2D NMR data, including the ^1^H–^1^H COSY, HSQC, and HMBC spectra (Fig. 2). The ^1^H–^1^H COSY spectrum revealed the connectivity of seven partial structures **a** (C-3, C-14–C-15), **b** (C-5–C-6), **c** (C-18–C-19), **d** (C-16'–C-17'), **e** (C-5'–C-6'), **f** (C-9'–C-12'), and **g** (C-18'–C-19') as shown in Fig. 2. These partial structures were classified into two units, A and B.

In unit A, the connectivity of partial structure **b** and the indoline ring (C-2, C-7–C-13 and N-1) was revealed by the HMBC correlations of H-6 and H-9 to C-7 (δ_C_ 54.3). HMBC correlations from H_3_-18 to C-20 (δ_C_ 44.2), H_2_-19 to C-15 (δ_C_ 131.3), H-17 to C-16 (δ_C_ 81.0), C-19 (δ_C_ 31.8) and C-21 (δ_C_ 67.4), and H-2 to C-21 established the connections among C-15, C-17, C-19, and C-21 through C-20 and the connection between C-21 and C-2 through C-7. HMBC cross peaks of H-21 to C-3 (δ_C_ 51.9) and C-5 (δ_C_ 52.3) suggested the linkage between C-3, C-5 and C-21 through a nitrogen atom. The presence of an acetoxy and a methoxycarbonyl functions at C-17 and C-16 was confirmed by HMBC correlations from H-17 and H_3_-26 to C-25 (δ_C_ 172.4), and H-2, H-17 and H_3_-27 to C-22 (δ_C_ 173.4), respectively. These data were characteristic of an aspidosperma skeleton.

In unit B, the presence of an oxyindole ring (C-2', C-7'–C-13' and N-1') in the partial structure **f** and the connection with the partial structure **e** were revealed by the HMBC correlations of H-6'a to C-2' (δ_C_ 181.0), H-9' to C-7' (δ_C_ 62.3). HMBC cross-peaks of H-6'b, H_2_-17', and H-21' to C-3' (δ_C_ 154.8), H_2_-17' and H-19' to C-15' (δ_C_ 147.3), and H-19' to C-21' (δ_C_ 140.1) established the presence of a pyridine ring and the connections among the partial structures **d** and **g**. The connectivity of C-10 in unit A and C-16' in **d** was deduced by the HMBC correlation of H_2_-17' to C-10 (δ_C_ 117.0). The presence of a methoxycarbonyl group at C-16' was indicated by the HMBC correlations of H_3_-23' and H_2_-17' to C-22' (δ_C_ 174.9). These data implied unit B possessed a modified iboga skeleton with the tetracyclic spirooxindole moiety produced by cleavage between C-2' and C-16'.

**Fig. 3:**
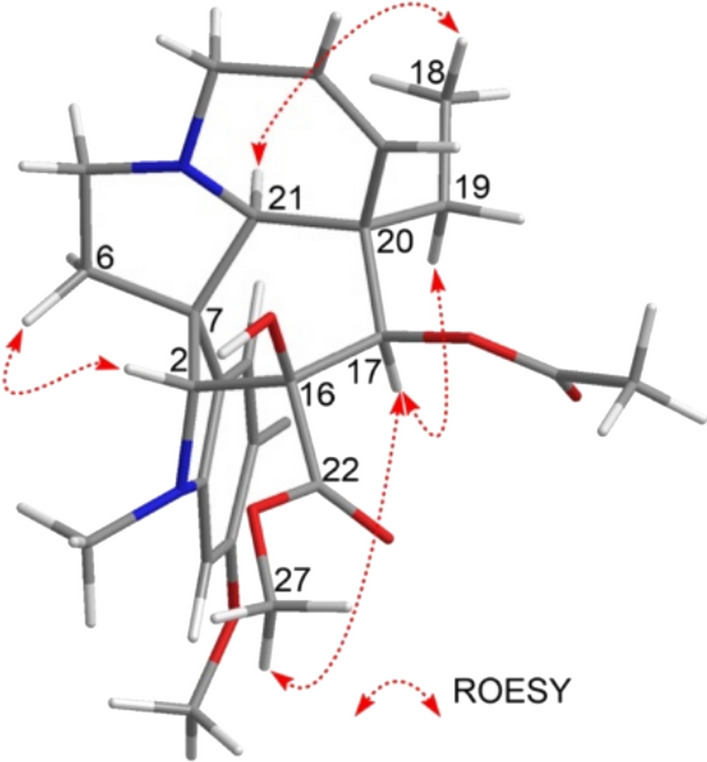
Selected ROESY correlations for unit A in cathagine A (**1**)

**Fig. 4:**
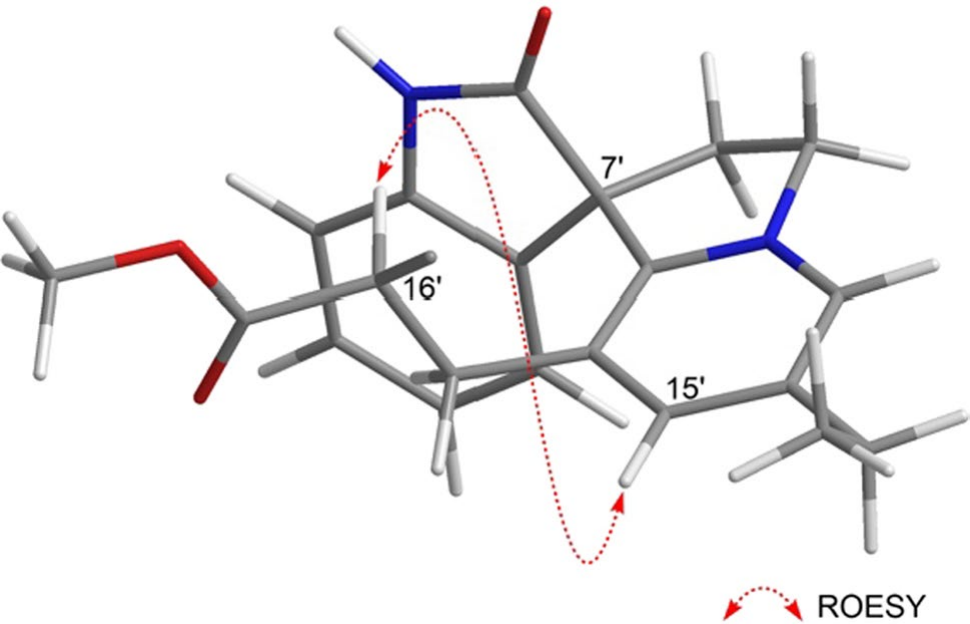
Selected ROESY correlations for unit B in cathagine A (**1**)

The relative configuration of each monoterpene indole unit in **1** was assigned by ROESY correlations as shown in computer-generated 3D drawing (Figs. 3 and 4).

In unit A (Fig. 3), ROESY correlations of H-17/H_2_-19 and H_3_-27, and H-21/H_3_-18 suggested that H-17, H-21, and C-22 were in the α-configuration. ROESY correlations of H-2/H-6 suggested that H-2 was estimated to be in the β-configuration. These ROE relationships were also found in a series of cathagines B-D (**2**–**4**) and the same as those in vindoline. On the other hand, in unit B, although ROESY correlations of H-15'/H-16' was observed, useful information to support the stereochemistry at C-7' was not obtained (Fig. 4).

**Fig. 5:**
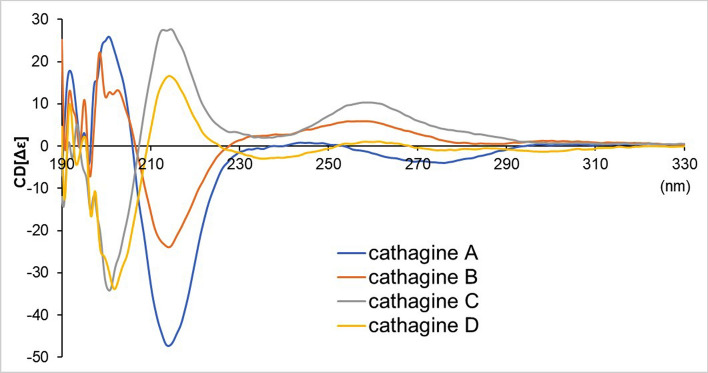
CD spectra for cathagines A–D (**1**–**4**)

The absolute configuration of cathagines A–D (**1**–**4**) was then assigned by comparing the CD spectra with those of the reported oxindole alkaloids [19–22]. As shown in Fig. 5, the CD spectra of cathagines A and B (**1** and **2**) show a similar CD pattern with that of rhynchophylline [21] with C-7*R* [20, 22], indicating that the spiro center at C-7' in unit B have the *R* configuration. On the other hand, the CD spectra of **3** and **4** showed a different CD pattern from those of **1** and **2**, which deduced to possess the *S* configuration of the spiro carbon at C-7' in unit B as in that of isorhynchophylline [20, 22].

The configuration at C-16' of cathagines A–D (**1**–**4**) was deduced by ROESY correlations. Each stable conformation of 7'*R*16'*R,* 7'*R*16'*S,* 7'*S*16'*R*, and 7'*S*16'*S* was obtained using Monte Carlo analysis with MMFF94 force field and charges on Macromodel 9.1 [23].

**Fig. 6:**
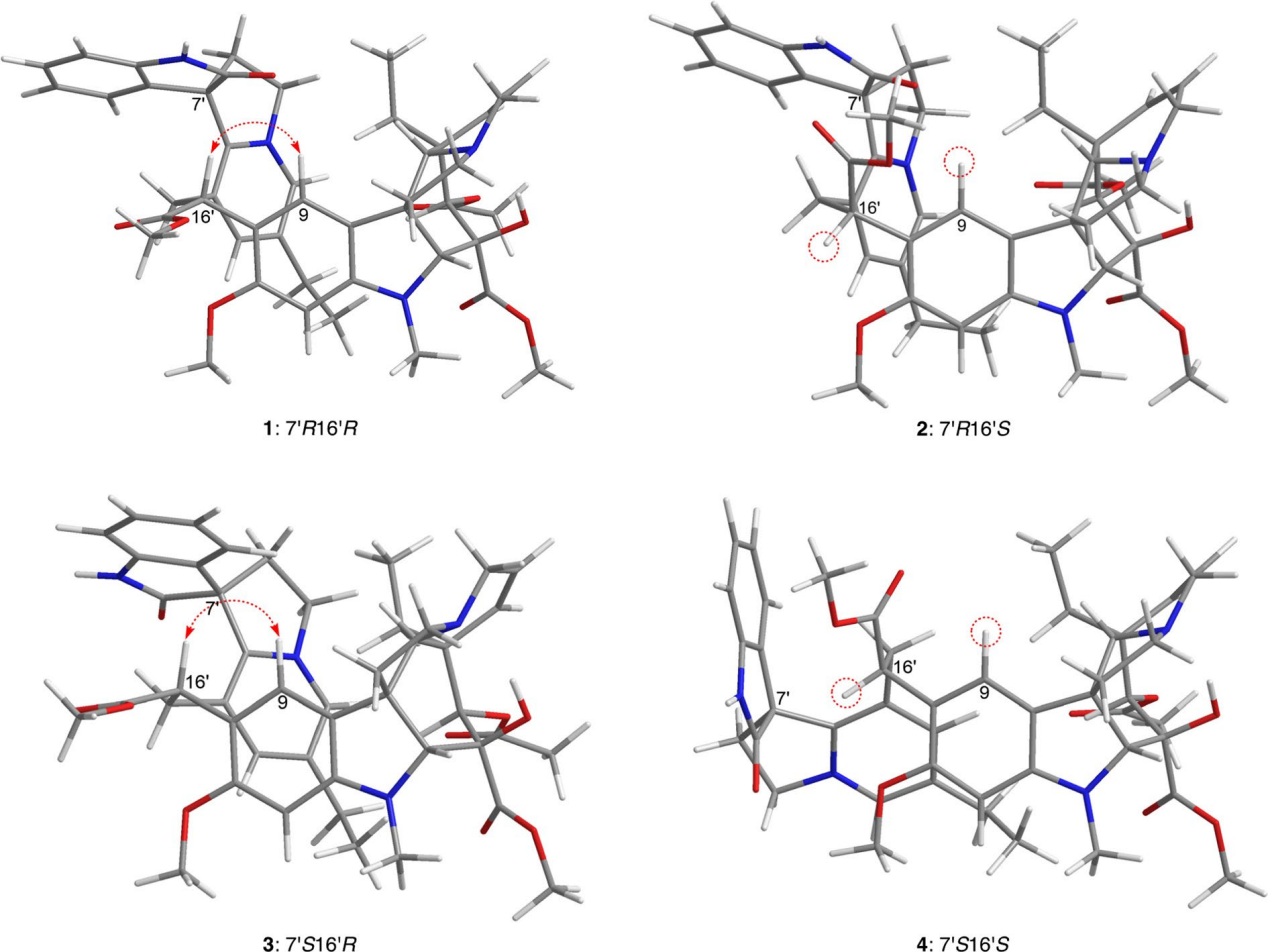
Each stable conformation for **1**–**4** and ROESY correlations between H-16’ and H-9 of **1** and **3**

Each ROESY correlation observed at H-9/H-16' of **1** and **3** supported the stereochemistry of C-16' is *R*, on the other hand, none of them of **2** and **4** that of C-16' is *S* (Fig. 6). Thus the absolute configuration of cathagines A–D (**1**–**4**) was deduced to be 2*R,*7*R*,16*S,*17*R,*20*R,*21*R,*7'*R,*16'*R* for **1**, 2*R,*7*R*,16*S,*17*R*,20*R,*21*R,*7'*R,*16'*S* for **2**, 2*R,*7*R*,16*S,*17*R*,20*R,*21*R,*7'*S*,16'*R* for **3**, and 2*R,*7*R* 16*S*,17*R,*20*R,*21*R,*2*S,*7'*S*,16'*S* for **4***.*

**Scheme 1.**
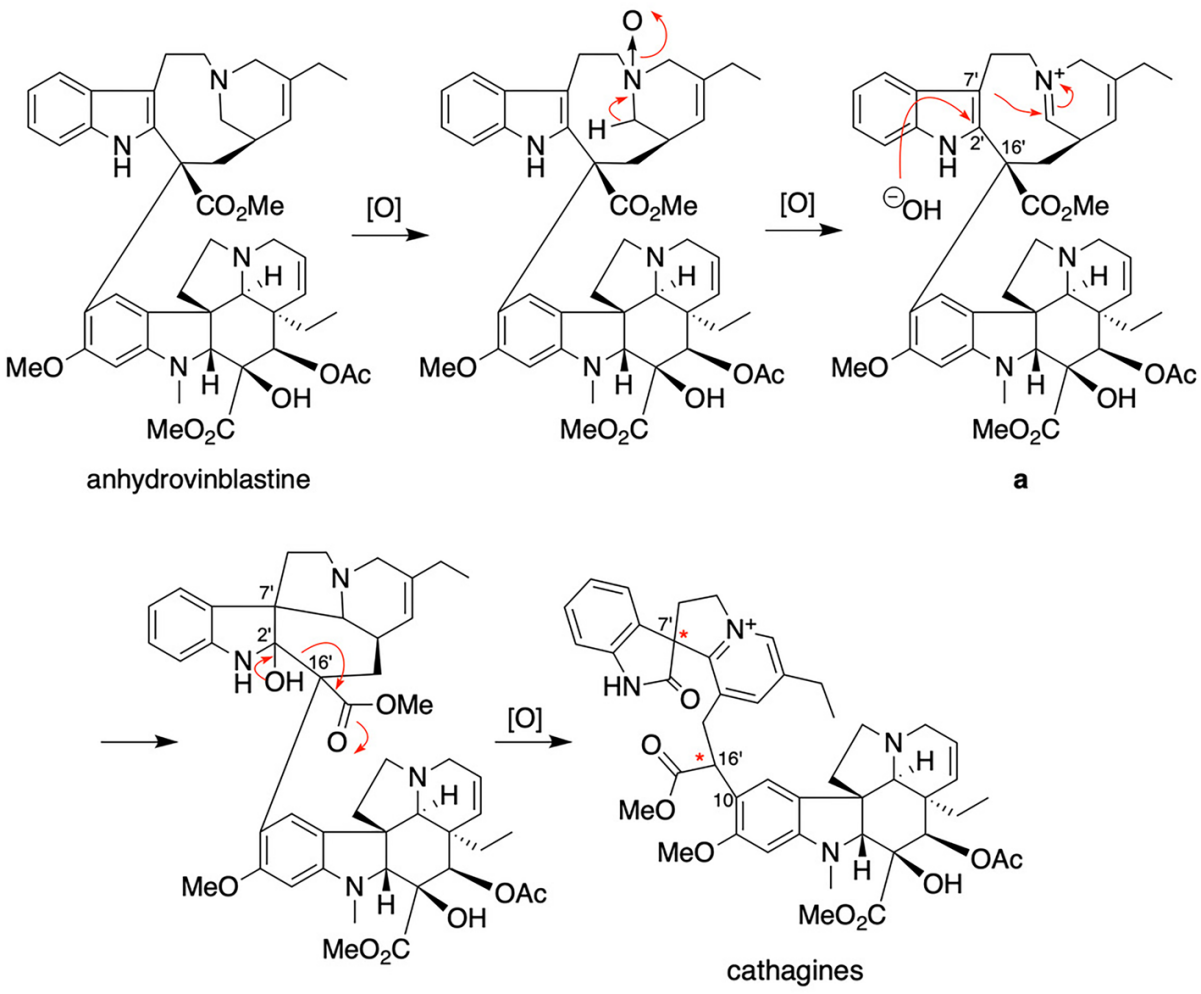
A plausible biogenetic path of cathagines A–D (**1**–**4**)

A plausible biogenetic path for cathagines A (**1**)–D (**4**) is proposed as shown in Scheme 1. A series of cathagines with the tetracyclic spirooxindole moiety might be derived by oxidative intramolecular coupling (C-7'–C-3') of imine intermediate **a** generated from the corresponding alkaloid such as anhydrovinblastine [24], followed by cleavage of the C-2'–C-16' bond of an iboga skeleton and oxidation into pyridine ring. Namely, it is thought to form a modified iboga skeleton without C-2'–C-16' bond though coupling by the Polonovski reaction [25] and bond cleavage by enolation.

### Biological activity of cathagines A (1)–D (4)

For *P. falciparum* 3D7 strain, cathagine B (**2**) with 7'*R*,16'*S* showed a moderate in vitro antimalarial activity [Table 3: the half-maximal (50%) inhibitory concentration (IC_50_) = 27.1 μM]. The cytotoxic activity of cathagine B (**2**) was low for HepG2 cells. These results showed that the two asymmetric carbon positions at C-7' and C-16' affect the activity.

**Table 3:** Antimalarial activity and cytotoxicity of cathagines A–D (**1**–**4**)

	3D7		HepG2	
	IC_50_ (μM)	Growth inhibition (%) 50 µM	Cytotoxicity (%) 50 µM	CC_50_(μM)
**1**	25.8		13.8	
**2**	27.1		4.4	
**3**	–	12.5%	19.8	
**4**	34.3		24.0	
Chloroquine	0.067			20.0

## Experimental section

### General experimental procedures

Optical rotations were measured on a JASCO DIP-1000 polarimeter. UV spectra were recorded on a Shimadzu UVmini-1240 spectrophotometer and IR spectra on a JASCO FT/IR-4100 spectrophotometer. High-resolution ESI MS were obtained on a JMS-T100LP (JEOL). ^1^H and 2D NMR spectra were measured on a 600 MHz spectrometer with cryoprobe at 300 K, while ^13^C NMR spectra were on a 150 MHz spectrometer. The residual solvent peaks were used as internal standards (*δ*_H_ 3.31 and *δ*_C_ 49.0 for CD_3_OD). Standard pulse sequences were used for the 2D NMR experiments. Merck silica gel 60 (40–63 μm) was used for the column chromatography, and the separations were monitored by Merck silica gel 60 F254, or Merck silica gel RP C-18 F254 TLC plates.

### Plant material

The plants of *Catharanthus roseus*, cultivated in the Tamil region of India were purchased in August 2020. The botanical identification was made by Mae Chu Corporation, Nara, Japan. Voucher specimens (Herbarium No. HU 4976) are deposited in the Herbarium of Hoshi University.

### Extraction and isolation

The whole dry plants of *Catharanthus roseus*, (10 kg) were extracted with MeOH (3 × 25 L, each 24 h), and the extract (400 g) was treated with 3% tartaric acid (pH 2) and then partitioned with EtOAc. The aqueous layer was treated with saturated Na_2_CO_3_ (aq) to pH 10 and extracted with CHCl_3_ to give an alkaloidal fraction (13.2 g). The alkaloidal fraction was subjected to LH-20 column (MeOH) two times, followed by an amino silica gel column (Hexane/EtOAc, 1:1 → 1:2 and then CHCl_3_/MeOH, 20:1 → 1:10, MeOH/25%NH_4_OH, 10:1), and the fractions were further separated using silica gel colum (CHCl_3_/MeOH, 10:1 → 1:1) and Cholester HPLC (CH_3_CN/ TFA 0.1% aq., 28:72) to give cathagine A (**1**, 25 mg, 0.0003%), cathagine B (**2**, 24 mg, 0.0002%), cathagine C (**3**, 33 mg, 0.0003%), and cathagine D (**4**, 39 mg, 0.0004%).

### Cathagine A

Yellow solid; [α] _D_^23^ – 133 (*c* 1.0, MeOH); IR (Zn–Se) *ν*_max_ 3453, 1733, and 1690 cm^−1^; CD (MeOH) *λ*_max_ (Δ*ε*) 201 (+ 26.2), 214 (– 47.3), 244 (+ 0.7), and 276 (– 4.2) nm;** U**V (MeOH) *λ*_max_ (log *ε*) 211 (4.52), 258 (3.95), and 370 (2.86) nm; ^1^H and ^13^C NMR data, see Tables 1 and 2; ESIMS *m/z* 805 (M)^+^; HRESIMS *m*/*z* 805.3806 (M)^+^calcd. for C_46_H_53_N_4_O_9_.

### Cathagine B

Yellow solid; [α] _D_^23^ + 75° (c 1.0, MeOH); IR (Zn–Se) *ν*_max_ 3446, 1734, and 1691 cm^−1^; CD (MeOH) *λ*_max_ (Δ*ε*) 202 (+ 12.3), 214 (-23.9), 258 (+ 5.3), and 300 (+ 1.0) nm;** U**V (MeOH) *λ*_max_ (log *ε*) 210 (4.53), 259 (3.95), and 365 (2.96) nm; ^1^H and ^13^C NMR data, see Tables 1 and 2; ESIMS *m/z* 805 (M)^+^; HRESIMS *m*/*z* 805.3803 (M)^+^calcd. for C_46_H_53_N_4_O_9_.

### Cathagine C

Yellow solid; [α] _D_^23^ + 95° (c 1.0, MeOH); IR (Zn–Se) *ν*_max_ 3446, 1734, and 1690 cm^−1^; CD (MeOH) *λ*_max_ (Δ*ε*) 201 (– 34.2), 215 (+ 27.9), 236 (+ 2.4), and 258 (+ 10.1) nm;;** U**V (MeOH) *λ*_max_ (log *ε*) 211 (4.52), 257 (3.95), and 370 (2.89) nm; ^1^H and ^13^C NMR data, see Tables 1 and 2; ESIMS *m/z* 805 (M)^+^; HRESIMS *m*/*z* 805.3807 (M)^+^calcd. for C_46_H_53_N_4_O_9_.

### Cathagine D

Yellow solid; [α] _D_^23^ -143° (c 1.0, MeOH); IR (Zn–Se) *ν*_max_ 3446, 1731 and 1687 cm^−1^; CD (MeOH) *λ*_max_ (Δ*ε*) 202 (– 34.0), 214 (+ 16.2), 235 (– 3.0), 261 (+ 0.8), 275 (– 0.7), and 299 (– 1.0) nm;** U**V (MeOH) *λ*_max_ (log *ε*) 211 (4.53), 258 (3.95), and 370 (2.89) nm; ^1^H and ^13^C NMR data, see Tables 1 and 2; ESIMS *m/z* 805 (M)^+^; HRESIMS *m*/*z* 805.3806 (M)^+^calcd. for C_46_H_53_N_4_O_9_.

### Conformational search

The conformations were obtained using Monte Carlo analysis with MMFF94 force field and charges on Macromodel 9.1 (Schrödinger, Inc.).

### Parasite strain and culture

*P. falciparum* laboratory strain 3D7 was obtained from Prof. Masatsugu Kimura (Osaka City University, Osaka, Japan). For the assessment of antimalarial activity of the compounds in vitro, the parasites were cultured in Roswell Park Memorial Institute (RPMI) 1640 medium supplemented with 0.5 g/L L-glutamine, 5.96 g/L HEPES, 2 g/L sodium bicarbonate (NaHCO_3_), 50 mg/L hypoxanthine, 10 mg/L gentamicin, 10% heat-inactivated human serum, and red blood cells (RBCs) at a 3% hematocrit in an atmosphere of 5% CO_2_, 5% O_2_, and 90% N_2_ at 37 °C as previously described [26]. Ring-form infected RBCs were collected using the sorbitol synchronization technique [27]. Briefly, the cultured cells were collected by centrifugation at 840 g for 5 min at room temperature, suspended in a fivefold volume of 5% D-sorbitol (Nacalai Tesque) for 10 min at room temperature, and then were washed twice with RPMI 1640 medium to remove the D-sorbitol. The utilization of blood samples of healthy Japanese volunteers for the parasite culture was approved by the institutional review committee of the Research Institute for Microbial Diseases (RIMD), Osaka University (approval number: 22–3).

### Antimalarial activity

Ring-form-synchronized parasites were cultured with **1**–**4** at sequentially decreasing concentrations (50, 15, 5, 1.5, 0.5, 0.15, 0.05, and 0.015 µM) for 48 h for the flow cytometric analysis using an automated hematology analyzer, XN-30. The XN30 analyzer was equipped with a prototype algorithm for cultured falciparum parasites (prototype; software version: 01–03, (build 16)) and used specific reagents (CELLPACK DCL, SULFOLYSER, Lysercell M, and Fluorocell M) (Sysmex, Kobe, Japan) [28, 29]. Approximately 100 µL of the culture suspension diluted with 100 µL phosphate-buffered saline was added to a BD Microtainer MAP Microtube for Automated Process K_2_ EDTA 1.0 mg tube (Becton Dickinson and Co., Franklin Lakes, NJ, USA) and loaded onto the XN-30 analyzer with an auto-sampler as described in the instrument manual (Sysmex). The parasitemia (MI-RBC%) was automatically reported [28]. Then 0.5% dimethyl sulfoxide alone or containing 5 µM artemisinin was used as the negative and positive controls, respectively. The growth inhibition (GI) rate was calculated from the MI-RBC% according to the following equation:

$$ {\text{GI }}\left( \% \right)\, = \,{1}00{-}\left( {{\text{test sample}}{-}{\text{positive control}}} \right)/\left( {{\text{negative control}}{-}{\text{positive control}}} \right)\, \times \,{1}00 $$

The IC_50_ was calculated from GI (%) using GraphPad Prism version 5.0 (GraphPad Prism Software, San Diego, CA, USA) [29].

### Cytotoxic activity

HepG2 (JCRB1054) cell line was obtained from the Japanese Collection of Research Bioresources (JCRB, Osaka, Japan). The cells were cultured in Dulbecco's modified Eagle's medium [DMEM (1.0 g/L glucose) with L-glutamine and sodium pyruvate; Nacalai Tesque] supplemented with 10% (v/v) fetal bovine serum (FBS; Gibco-BRL, Grand Island, NY, USA) in a humidified incubator with 5% CO_2_ at 37 °C. For the cytotoxic assay, the cells (5 × 10^3^/well) were seeded in a 96-well plate. compounds were added to the cell culture after 24 h and the cells were subsequently cultured for 48 h. Cell viability was measured using a Cell Counting Kit-8 (Dojindo, Kumamoto, Japan) according to the manufacturer's instructions. Briefly, 10 µL CCK-8 reagent was added to each well-containing culture medium and incubated for 2 h under standard culture conditions. The absorbance of the sample was measured at 450 nm using a PowerWave HT microplate spectrophotometer (BioTek Instruments, Winooski, VT, USA). The cell viability was expressed as a percentage of the absorbance of the untreated control cells after subtracting the appropriate background intensity.

## Supplementary Information

Below is the link to the electronic supplementary material.

Supplementary file1 (PDF 7344 KB)
