# Supplementary material for: Cathagines A–D, new bisindole alkaloids from Catharanthus roseus
Source: J Nat Med. 2024 Nov 14;79(1):134–42. doi: 10.1007/s11418-024-01857-4 (PMC13303443; doi:10.1007/s11418-024-01857-4)
Supplement: Supplementary file 1 — Supplementary file1 (PDF 7344 KB) [file 11418_2024_1857_MOESM1_ESM.pdf]

Supporting Information

Cathagines A-D, new bisindole alkaloids from *Catharanthus roseus*

Yusuke Hirasawa[a],, Chiaki Kasagi[a], Erika Koyama[a], Hitomi Myojin[a], Takahiro Tougan[b], Toshihiro Horii[c], Nahoko Uchiyama[d], Toshio Kaneda[a], Hiroshi Morita[a],**

[a]*Faculty of Pharmaceutical Sciences, Hoshi University, Ebara 2-4-41 Shinagawa-ku, Tokyo 142-8501, Japan*
[b]*Research Center for Infectious Disease Control, Research Institute for Microbial Diseases, Osaka University, 3-1 Yamadaoka, Suita, Osaka 565-0871, Japan*
[c]*Department of Malaria Vaccine Development, Research Institute for Microbial Diseases, Osaka University, 3-1 Yamadaoka, Suita, Osaka 565-0871, Japan*
[d]*National Institute of Health Sciences, 3-25-26 Tonomachi, Kawasaki-ku, Kawasaki, Kanagawa 210-9501, Japan*

Supporting Information

Figure S1. ^{1}H NMR spectrum of cathagine A (**1**) in CD_3OD. S2
Figure S2. ^{13}C NMR spectrum of cathagine A (**1**) in CD_3OD. S3
Figure S3. HSQC spectrum of cathagine A (**1**) in CD_3OD. S4
Figure S4. ^{1}H-^{1}H COSY spectrum of cathagine A (**1**) in CD_3OD. S5
Figure S5. HMBC spectrum of cathagine A (**1**) in CD_3OD. S6
Figure S6. ROESY spectrum of cathagine A (**1**) in CD_3OD. S7
Figure S7. ^{1}H NMR spectrum of cathagine B (**2**) in CD_3OD. S8
Figure S8. ^{13}C NMR spectrum of cathagine B (**2**) in CD_3OD. S9
Figure S9. HSQC spectrum of cathagine B (**2**) in CD_3OD. S10
Figure S10. ^{1}H-^{1}H COSY spectrum of cathagine B (**2**) in CD_3OD. S11
Figure S11. HMBC spectrum of cathagine B (**2**) in CD_3OD. S12
Figure S12. ROESY spectrum of cathagine B (**2**) in CD_3OD. S13
Figure S13. ^{1}H NMR spectrum of cathagine C (**3**) in CD_3OD. S14
Figure S14. ^{13}C NMR spectrum of cathagine C (**3**) in CD_3OD. S15
Figure S15. Dept 135 spectrum of cathagine C (**3**) in CD_3OD. S16
Figure S16. HSQC spectrum of cathagine C (**3**) in CD_3OD. S17
Figure S17. ^{1}H-^{1}H COSY spectrum of cathagine C (**3**) in CD_3OD. S18
Figure S18. HMBC spectrum of cathagine C (**3**) in CD_3OD. S19
Figure S19. ROESY spectrum of cathagine C (**3**) in CD_3OD. S20
Figure S20. ^{1}H NMR spectrum of cathagine D (**4**) in CD_3OD. S21
Figure S21. ^{13}C NMR spectrum of cathagine D (**4**) in CD_3OD. S22
Figure S22. HSQC spectrum of cathagine D (**4**) in CD_3OD. S23
Figure S23. ^{1}H-^{1}H COSY spectrum of cathagine D (**4**) in CD_3OD. S24
Figure S24. HMBC spectrum of cathagine D (**4**) in CD_3OD. S25
Figure S25. ROESY spectrum of cathagine D (**4**) in CD_3OD. S26

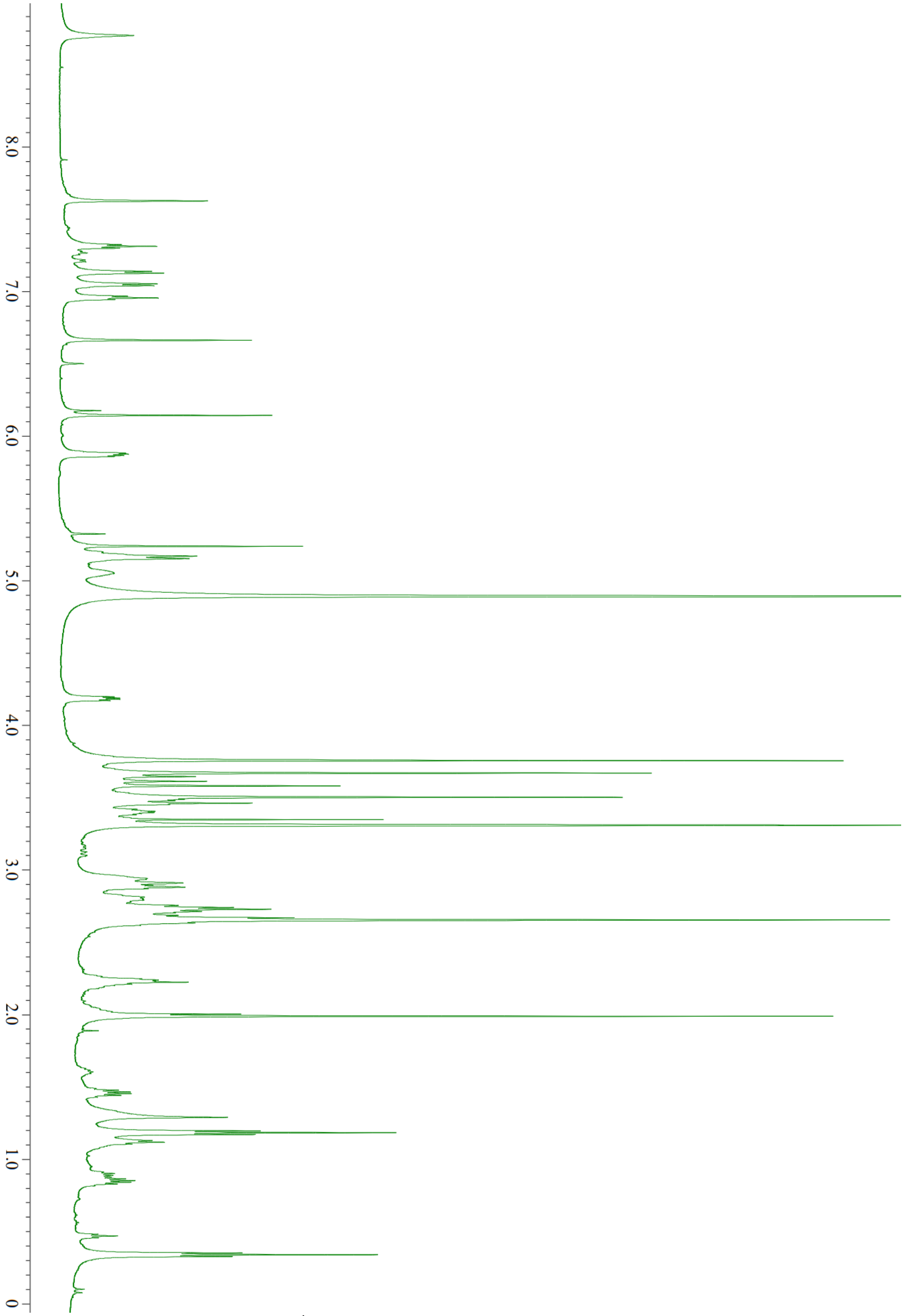

Figure S1. ^{1}H NMR spectrum of cathagine A (**1**) in CD_3OD.

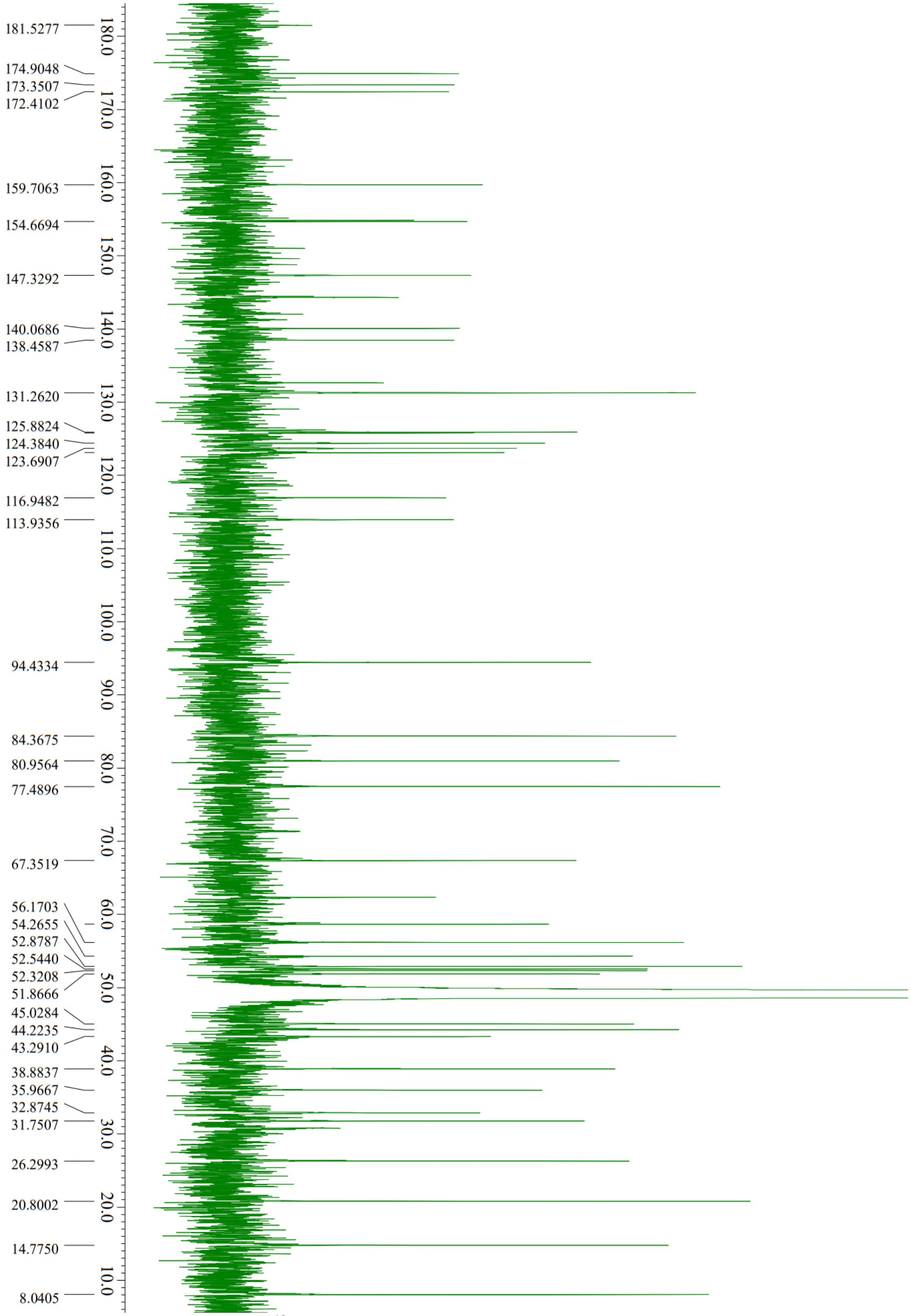

Figure S2. ^{13}C NMR spectrum of cathagine A (**1**) in CD_3OD.

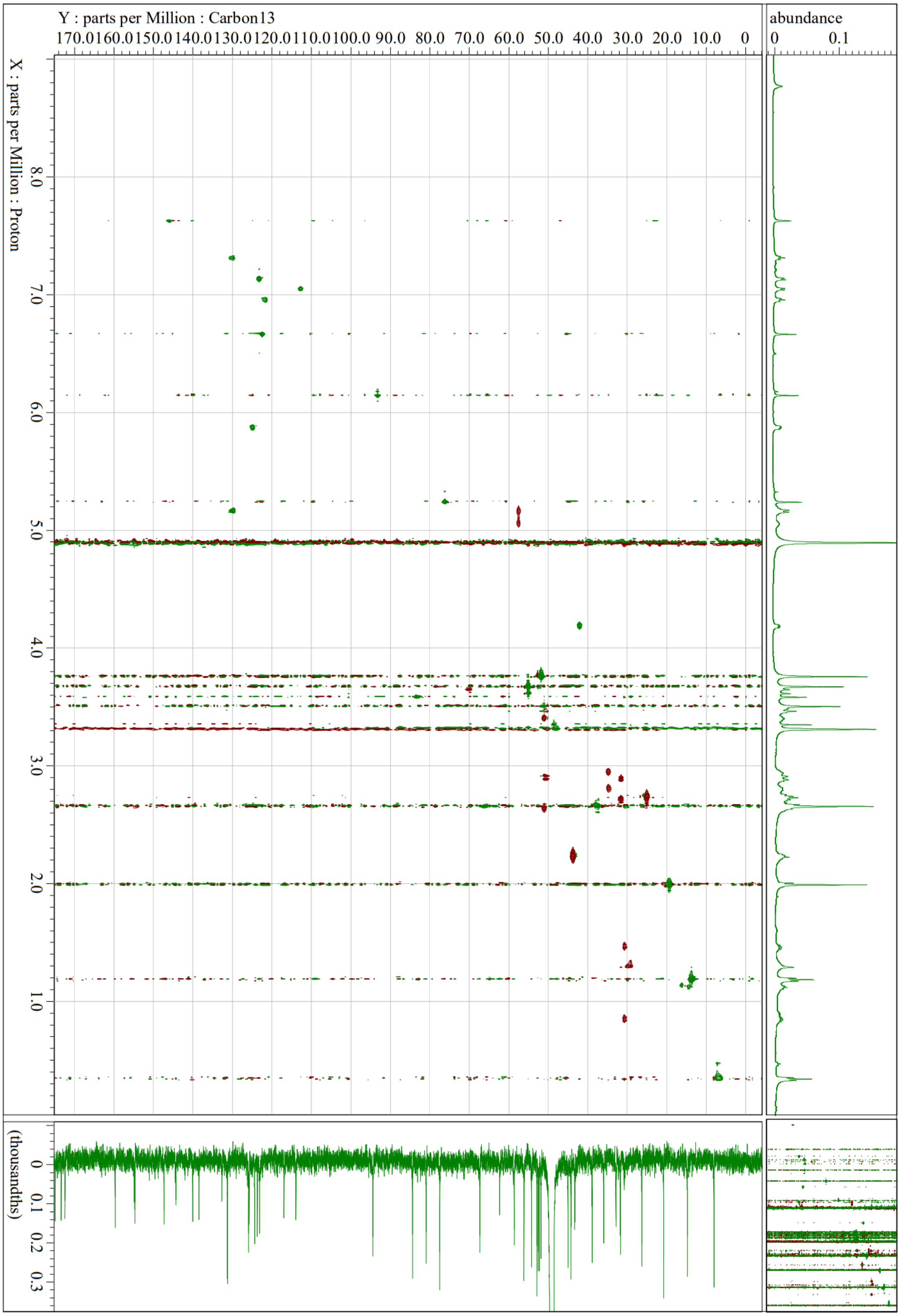

Figure S3. HSQC spectrum of cathagine A (1) in CD_3OD.

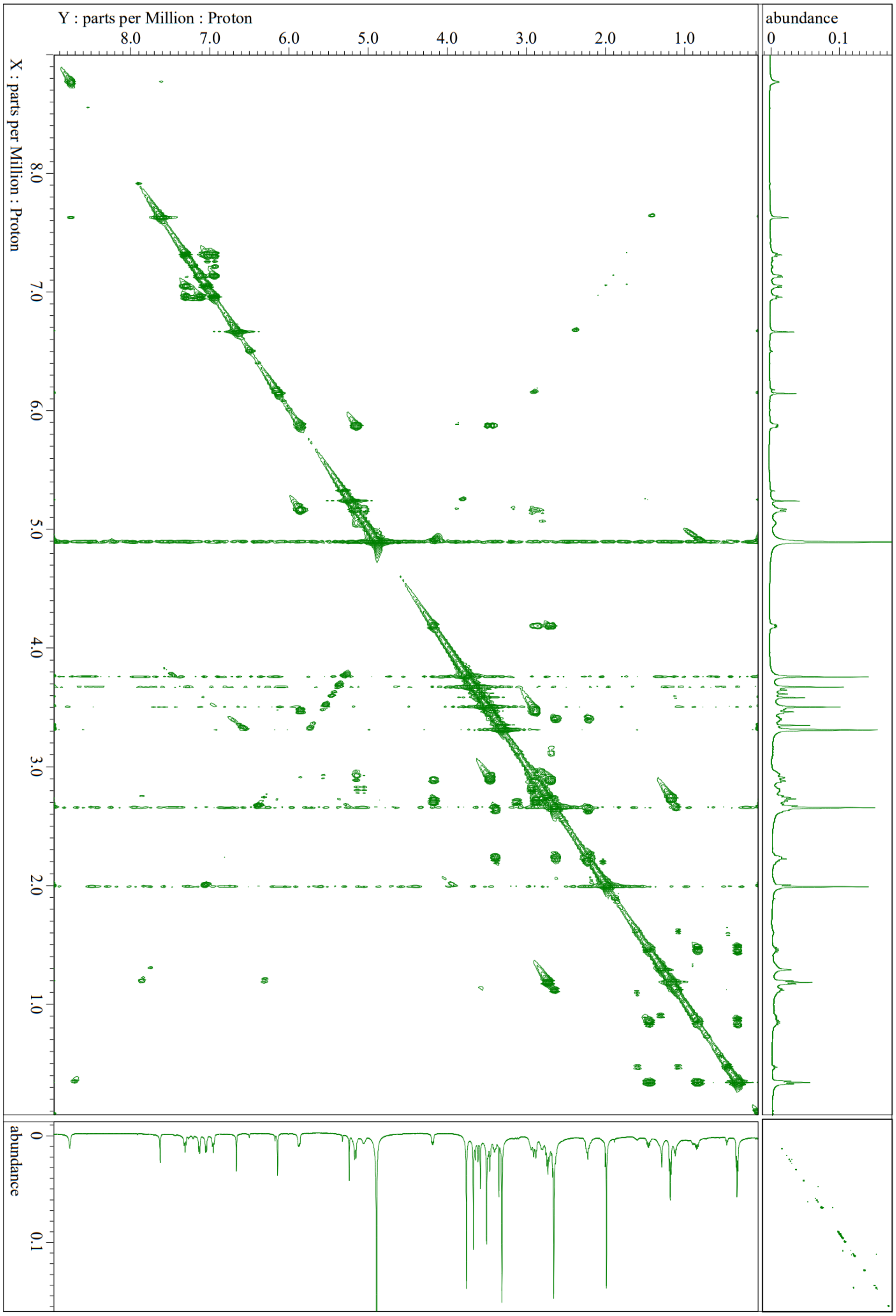

Figure S4. ^{1}H-^{1}H COSY spectrum of cathagine A (**1**) in CD_3OD.

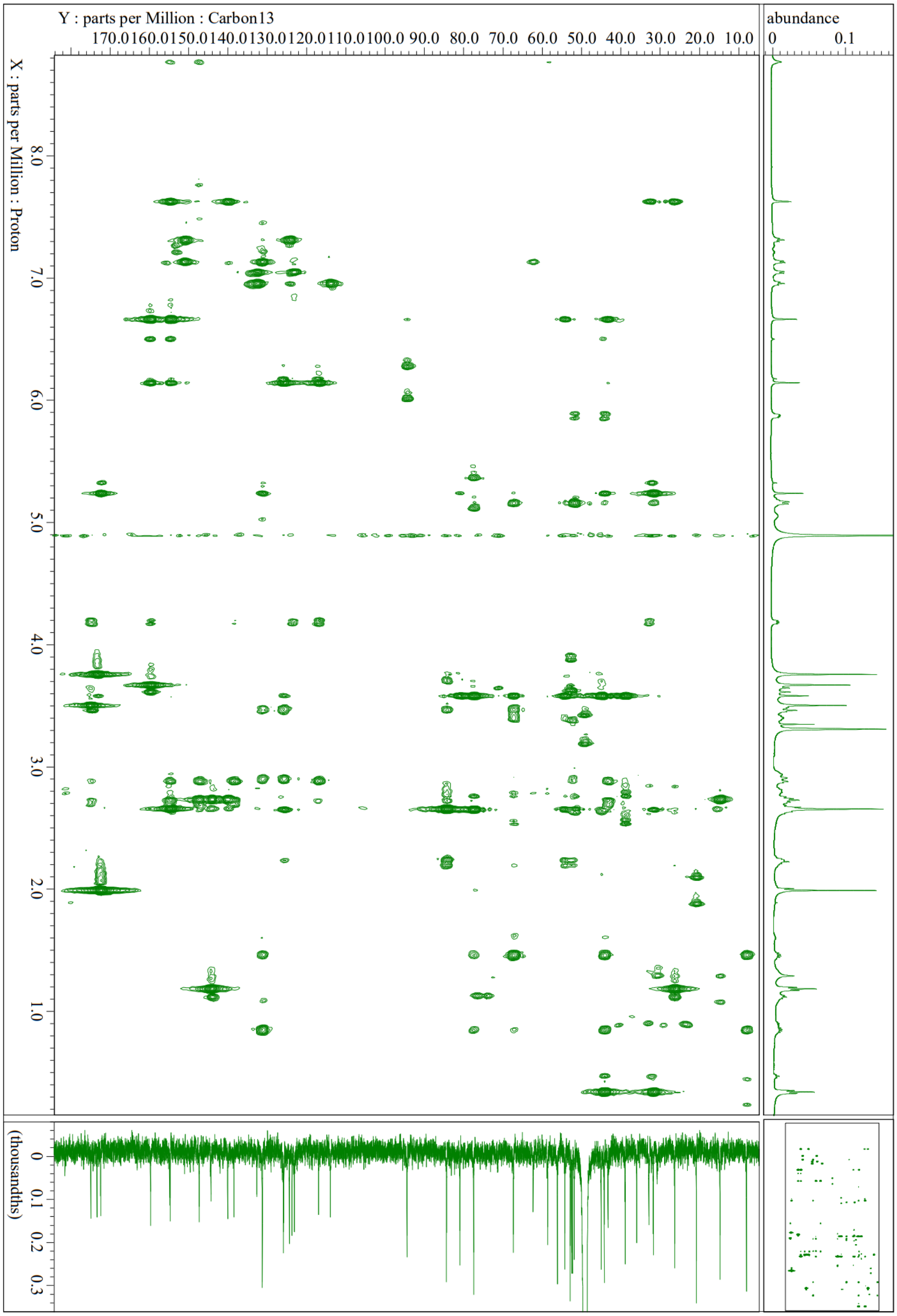

Figure S5. HMBC spectrum of cathagine A (**1**) in CD_3OD.

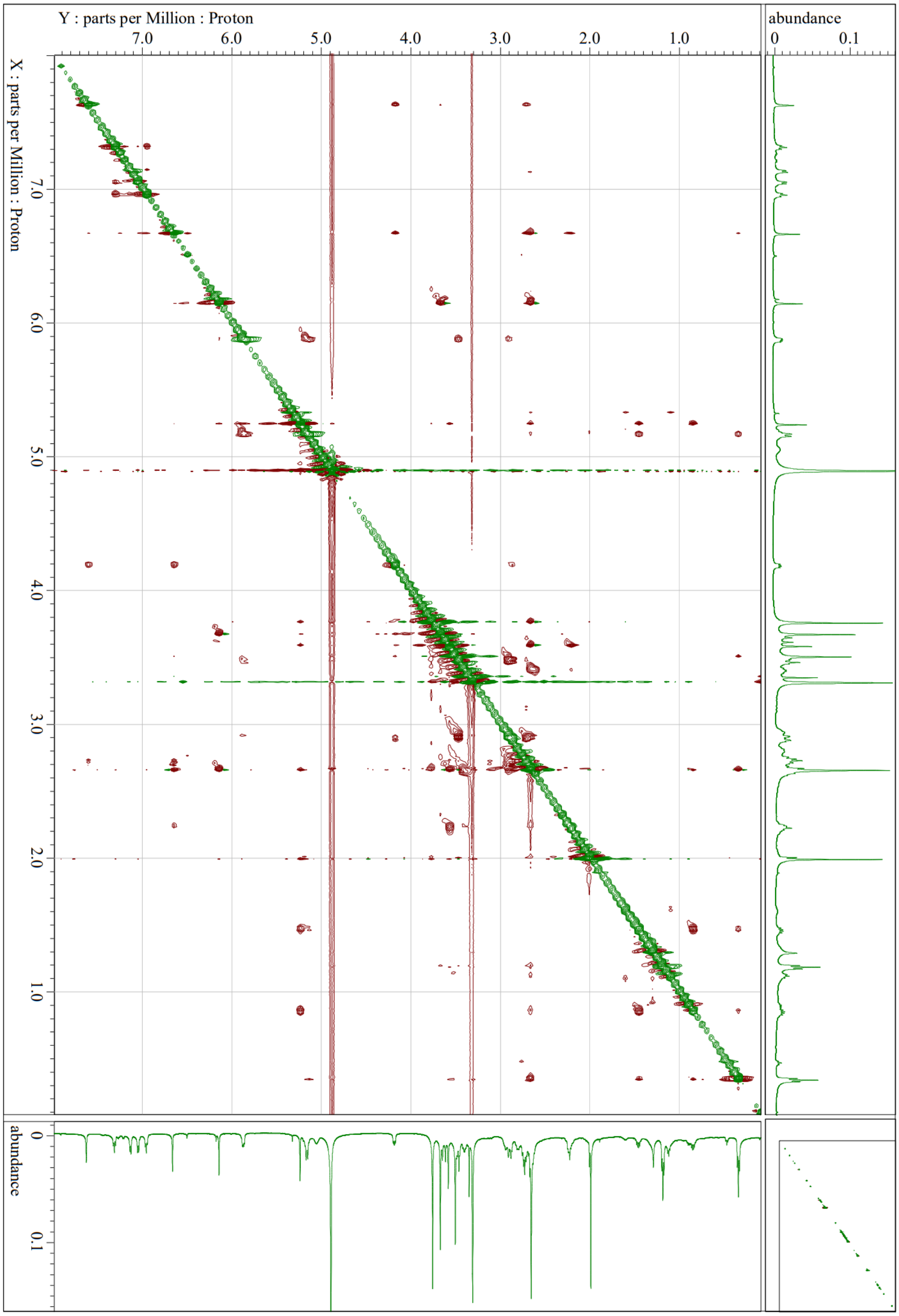

Figure S6. ROESY spectrum of cathagine A (**1**) in CD_3OD.

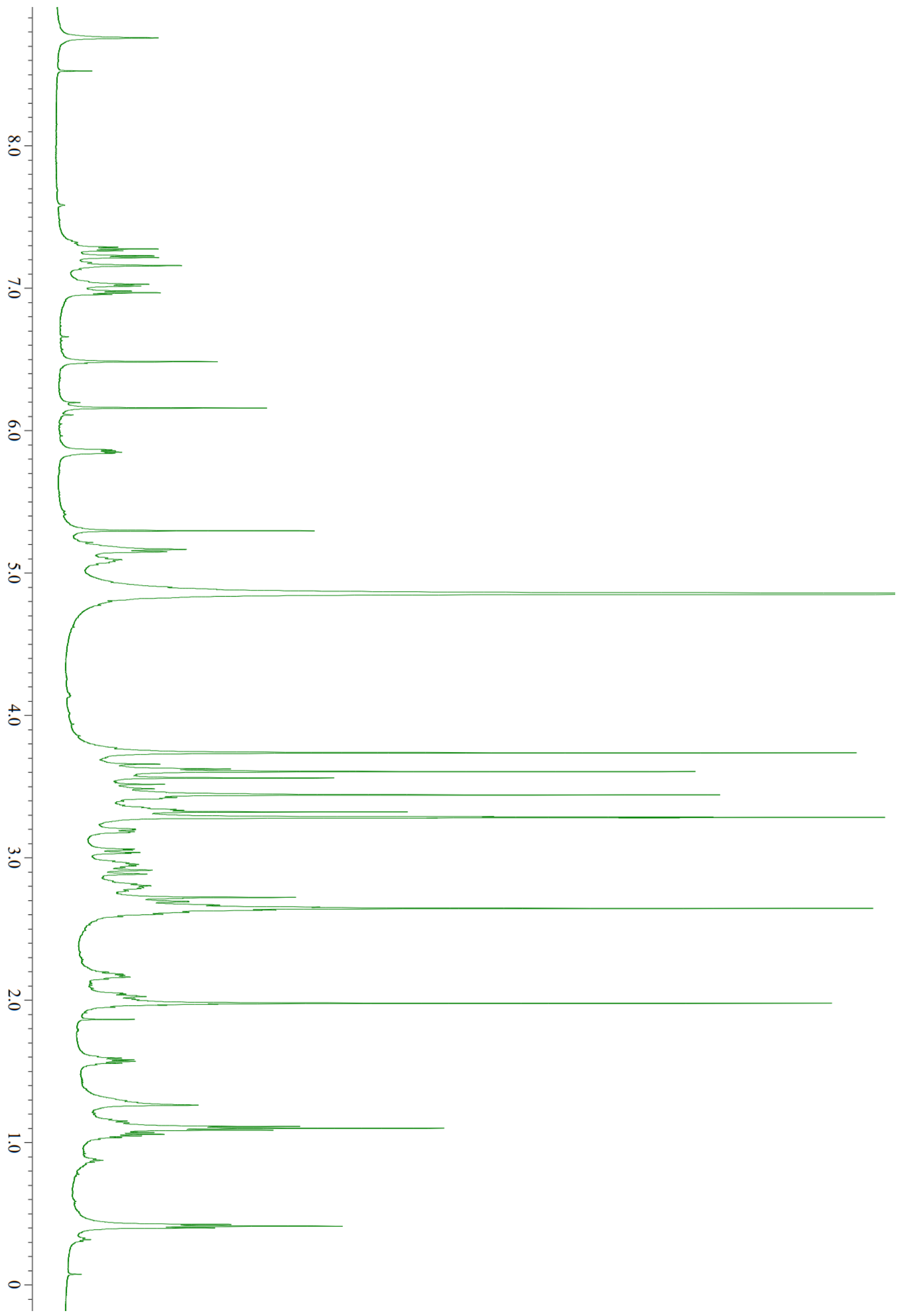

Figure S7. ^{1}H NMR spectrum of cathagine B (**2**) in CD_3OD.

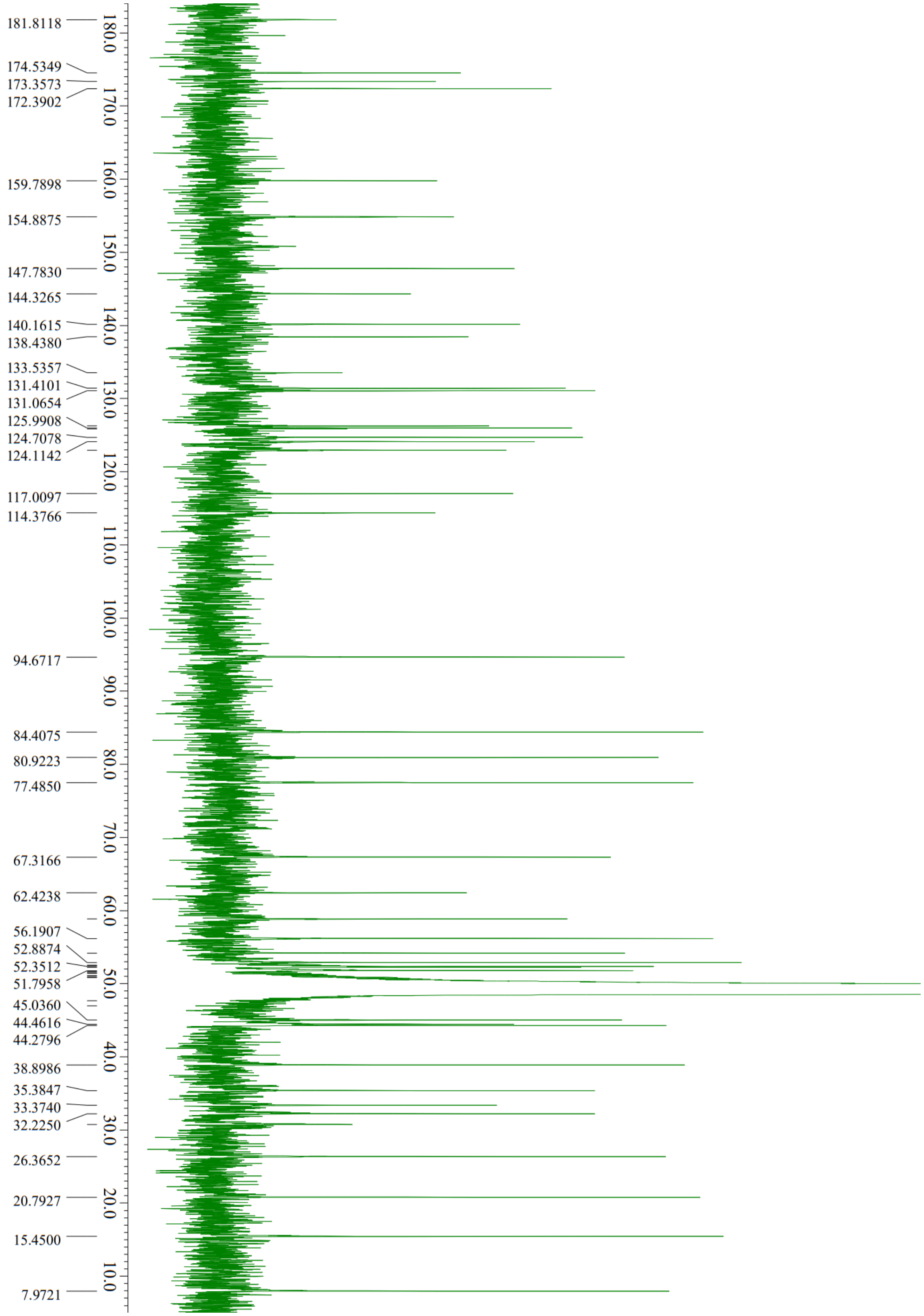

Figure S8. ^{13}C NMR spectrum of cathagine B (**1**) in CD_3OD.

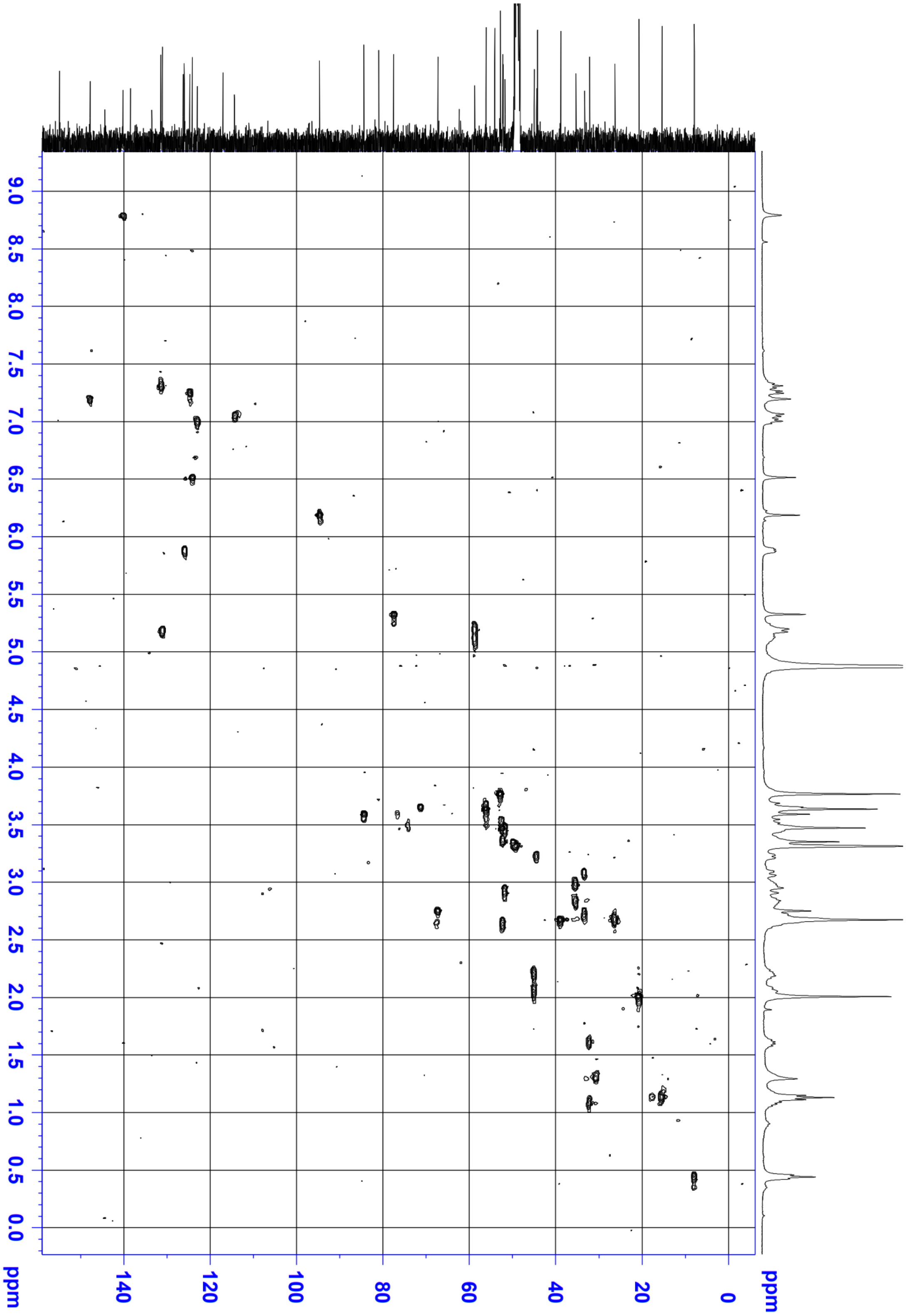

Figure S9. HSQC spectrum of cathagine B (2) in CD_3OD.

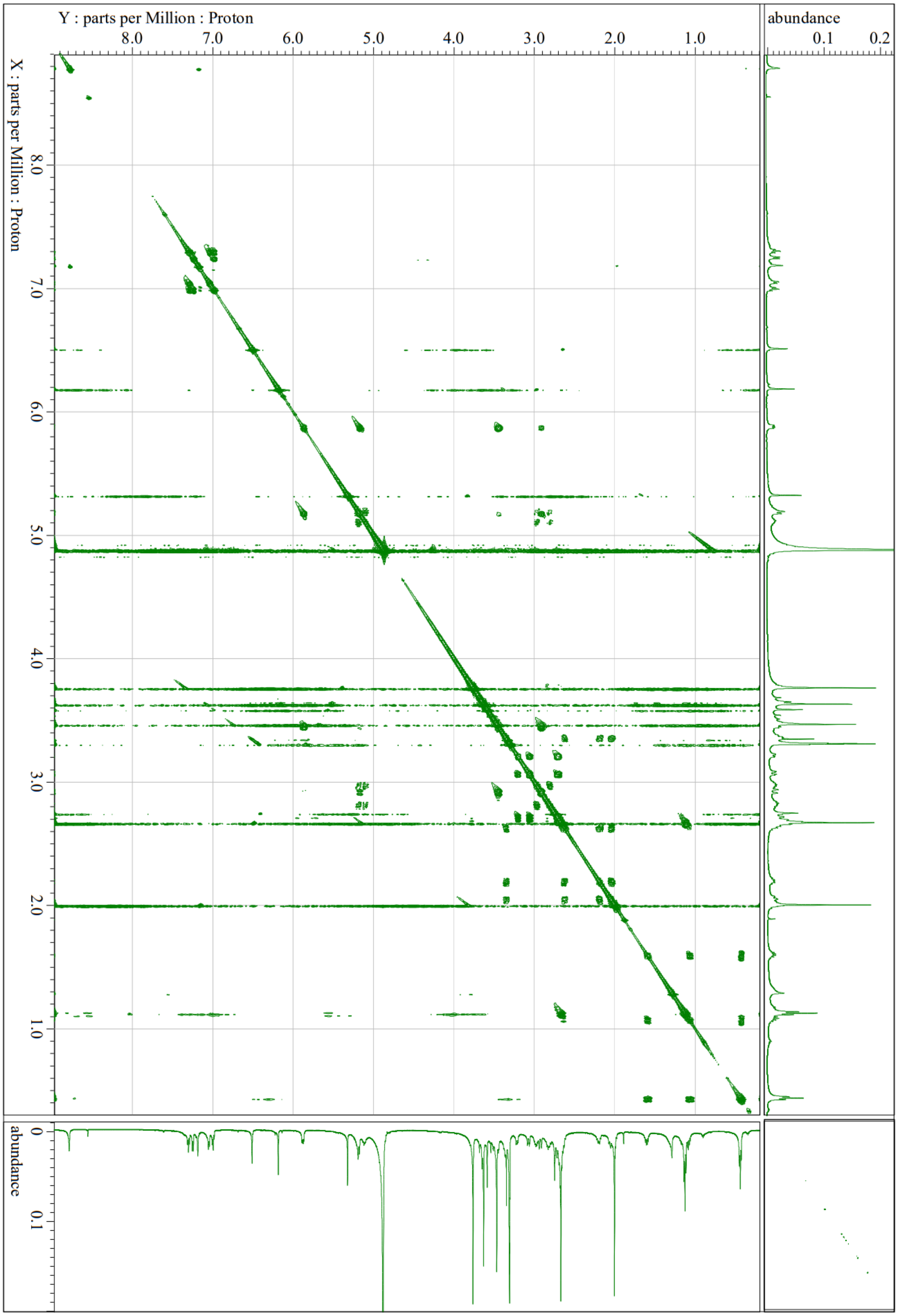

Figure S10. ^{1}H-^{1}H COSY spectrum of cathagine B (**2**) in CD_3OD.

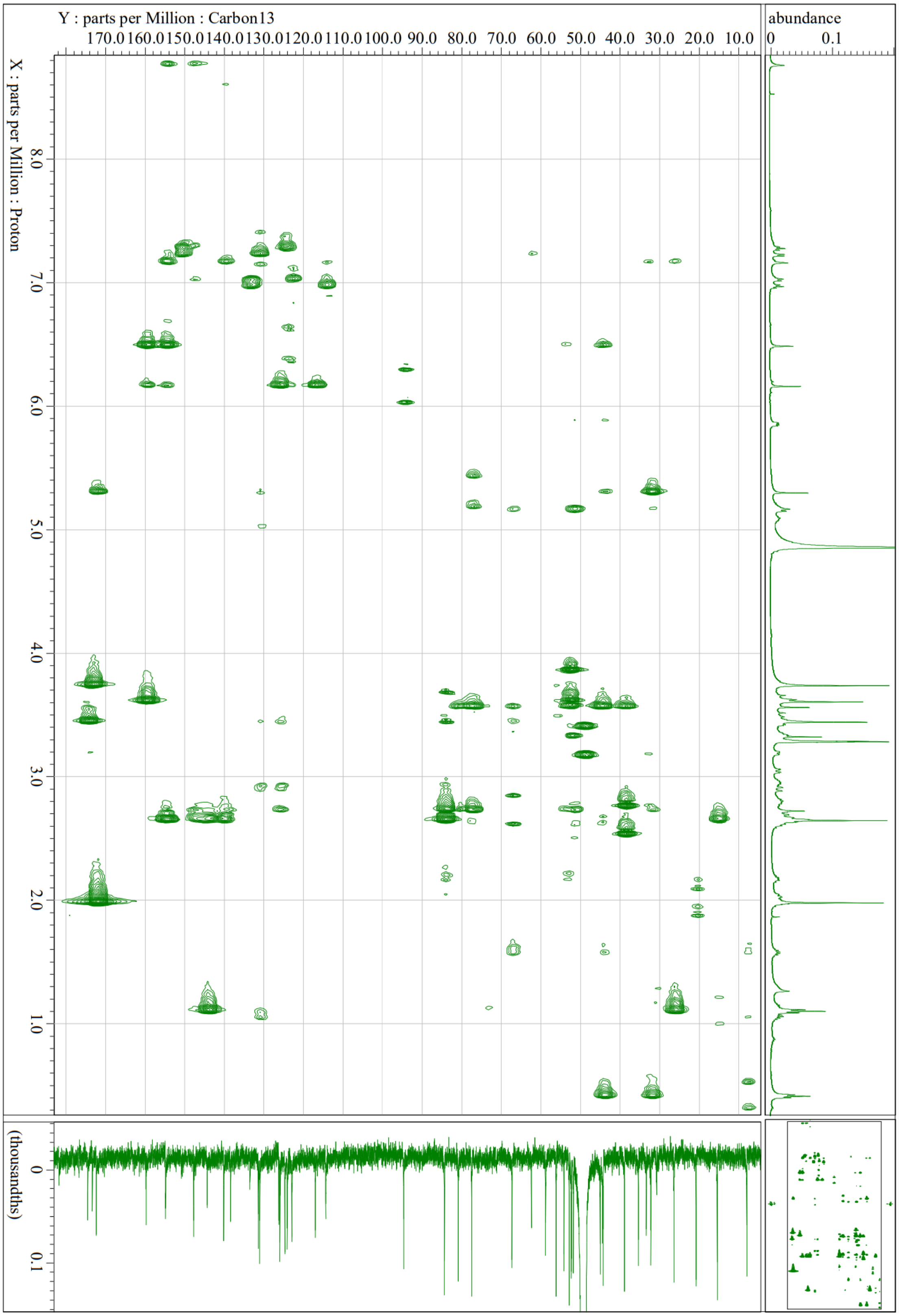

Figure S11. HMBC spectrum of cathagine B (**2**) in CD_3OD.

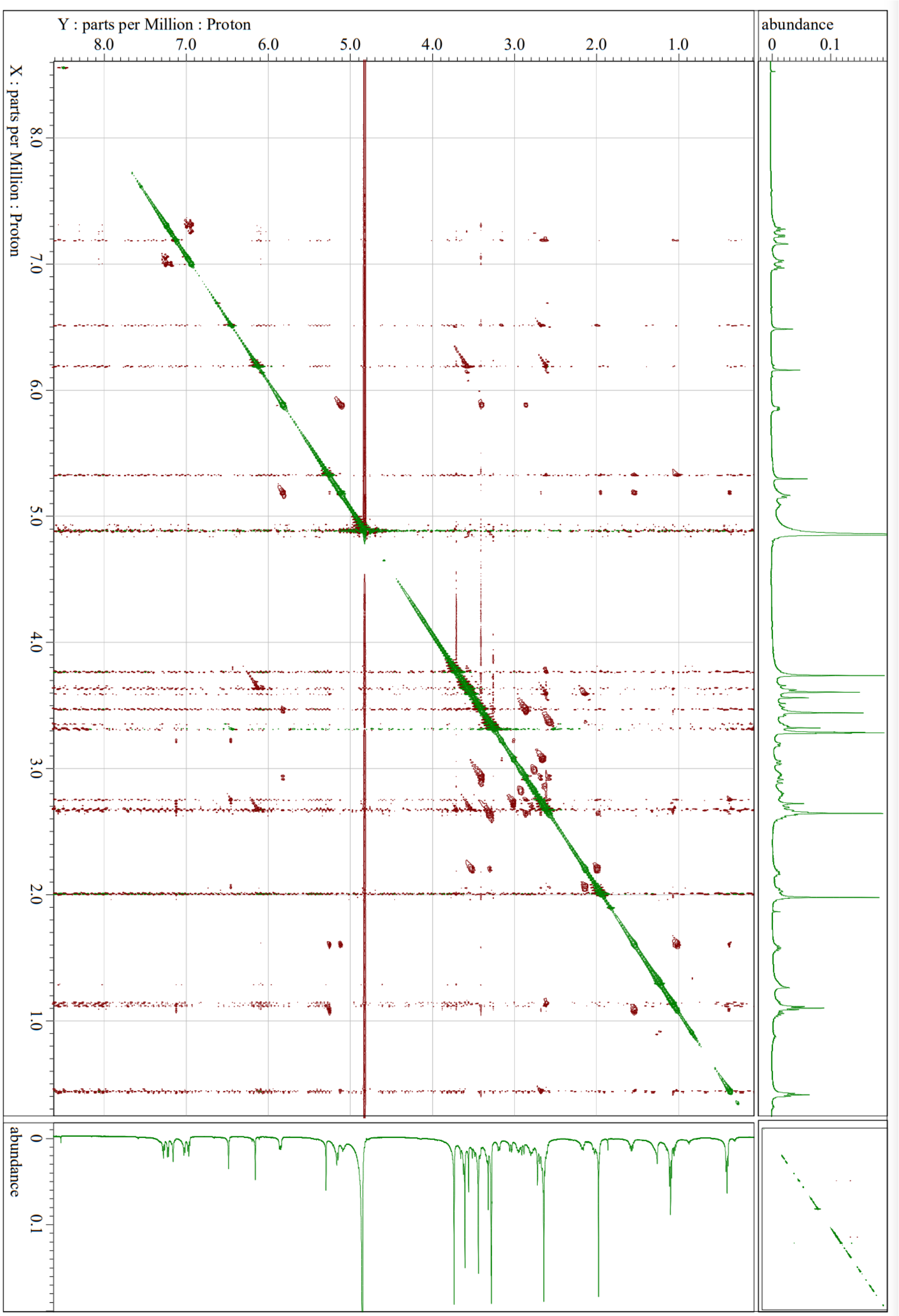

Figure S12. ROESY spectrum of cathagine B (**2**) in CD_3OD.

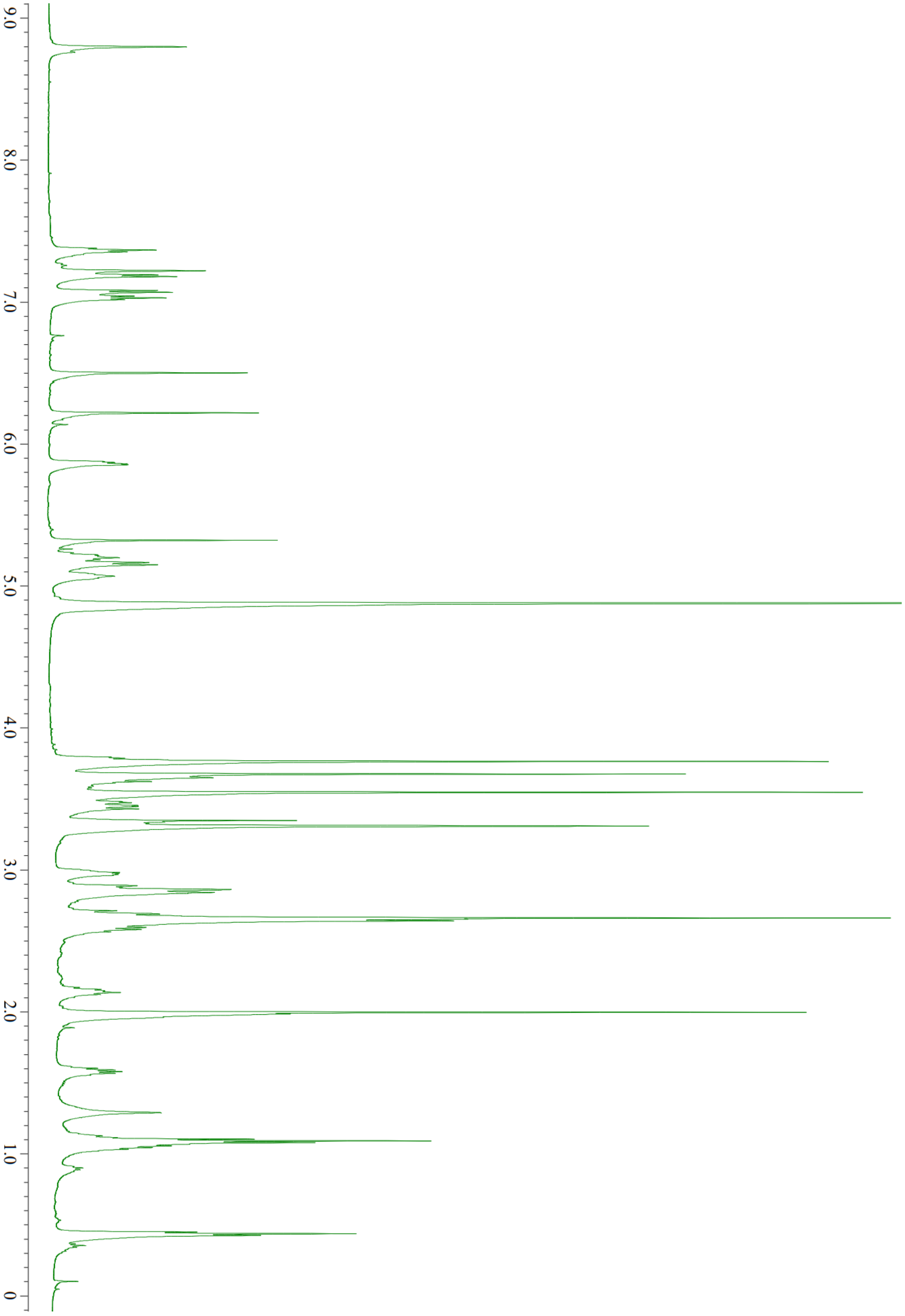

Figure S13. ^{1}H NMR spectrum of cathagine C (**3**) in CD_3OD.

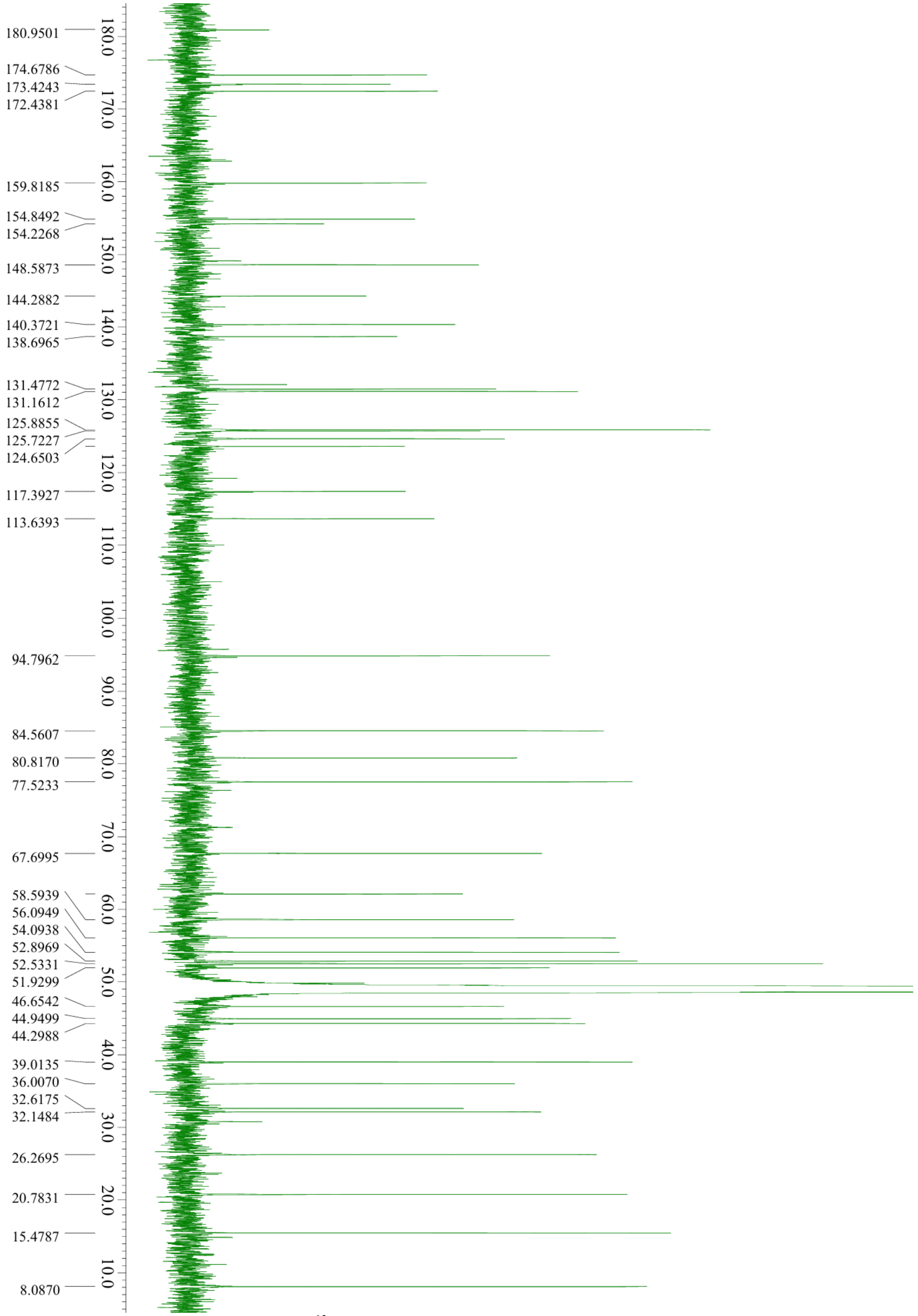

Figure S14. ^{13}C NMR spectrum of cathagine C (**3**) in CD_3OD.

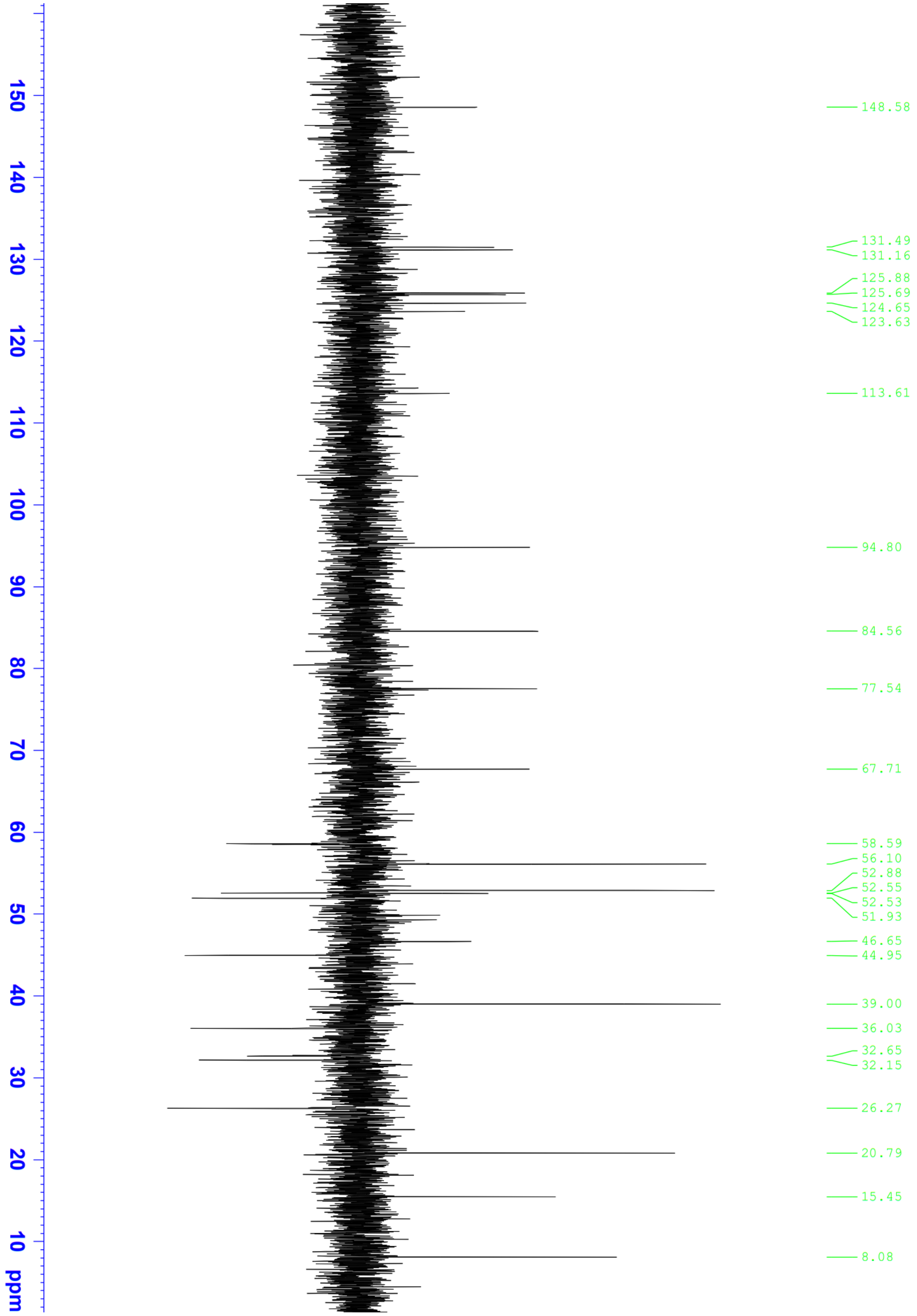

Figure S15. Dept 135 spectrum of cathagine C (**3**) in CD_3OD.

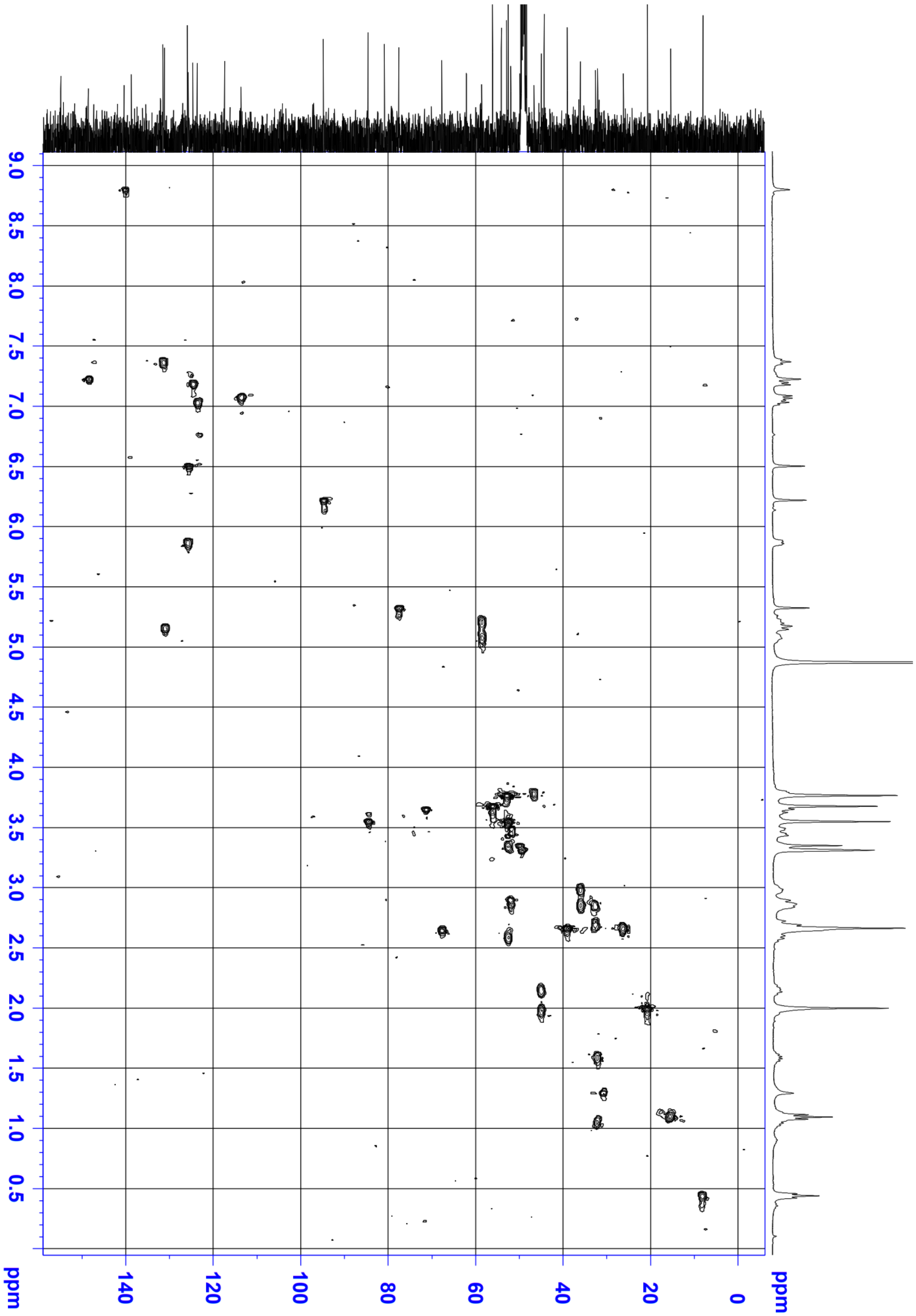

Figure S16. HSQC spectrum of cathagine C (**3**) in CD_3OD.

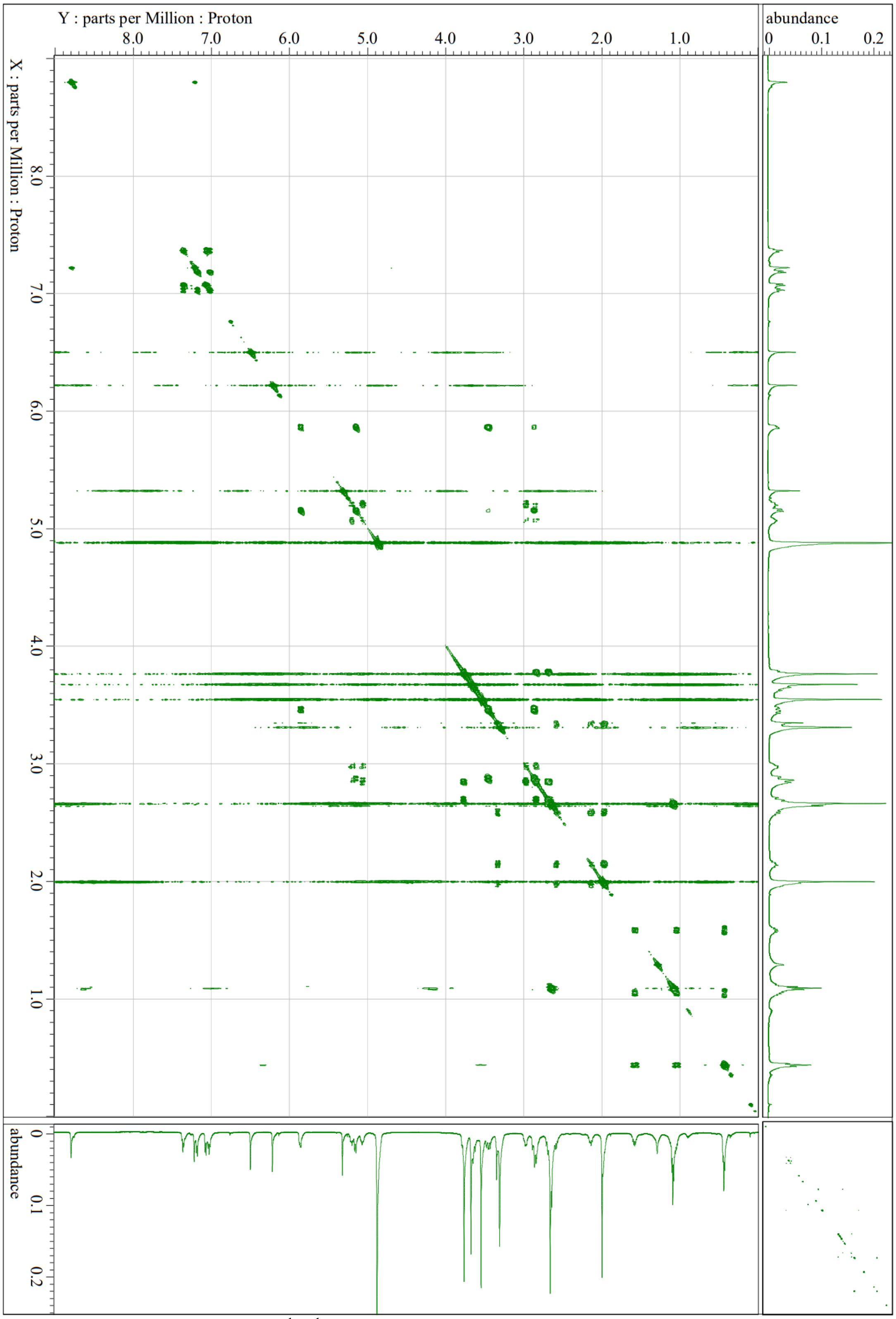

Figure S17. ^{1}H-^{1}H COSY spectrum of cathagine C (**3**) in CD_3OD.

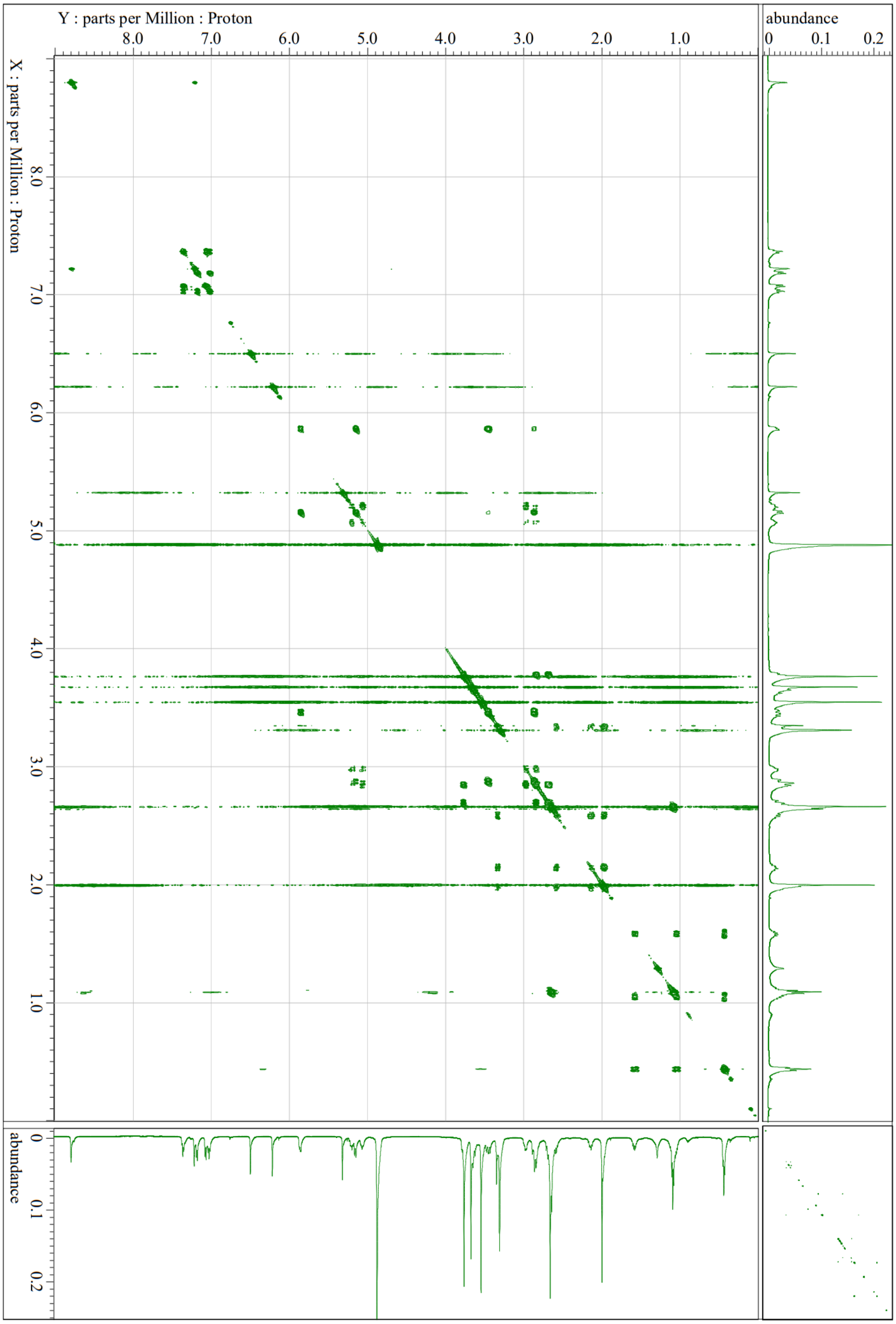

Figure S18. HMBC spectrum of cathagine C (**3**) in CD_3OD.

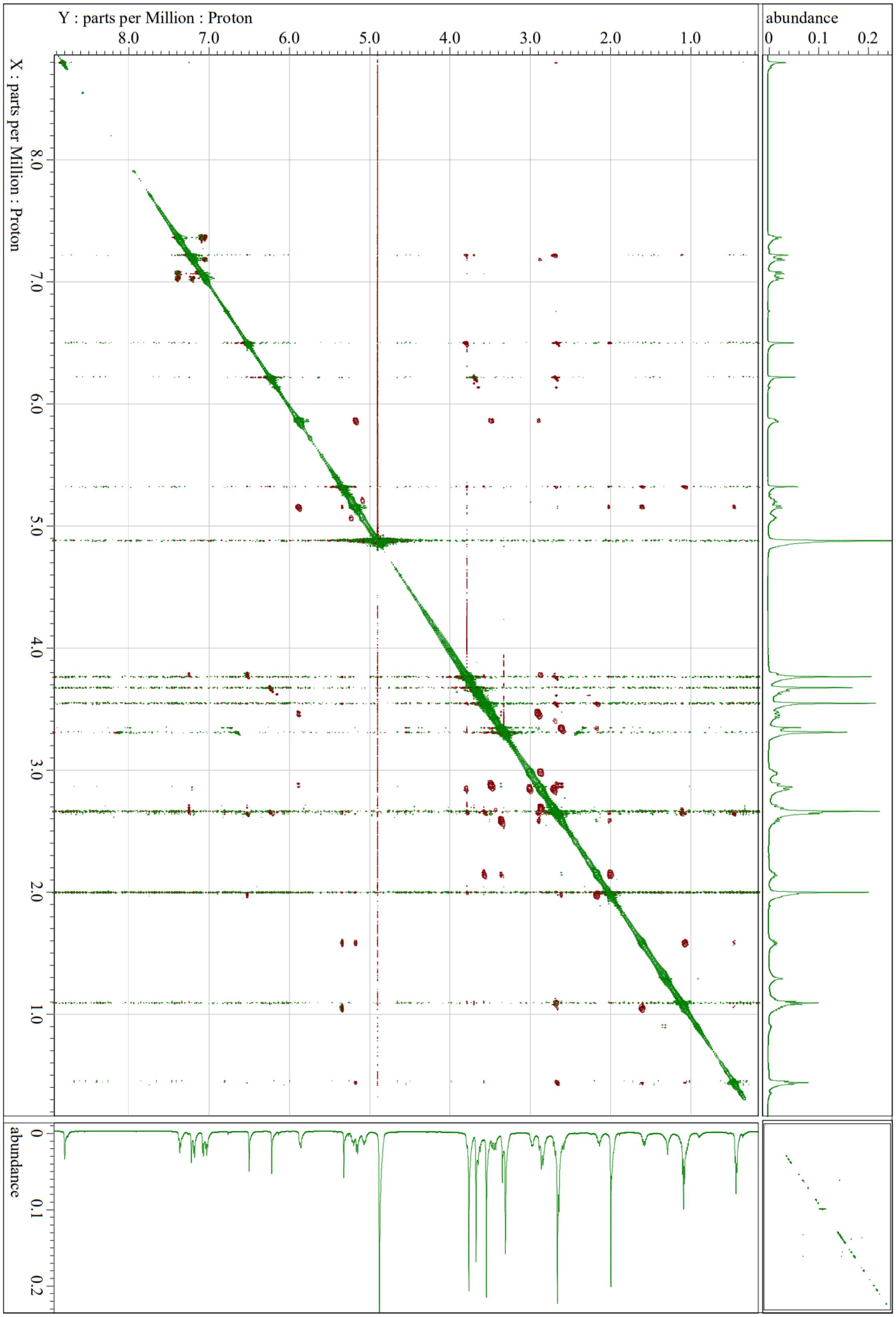

Figure S19. ROESY spectrum of cathagine C (**3**) in CD_3OD.

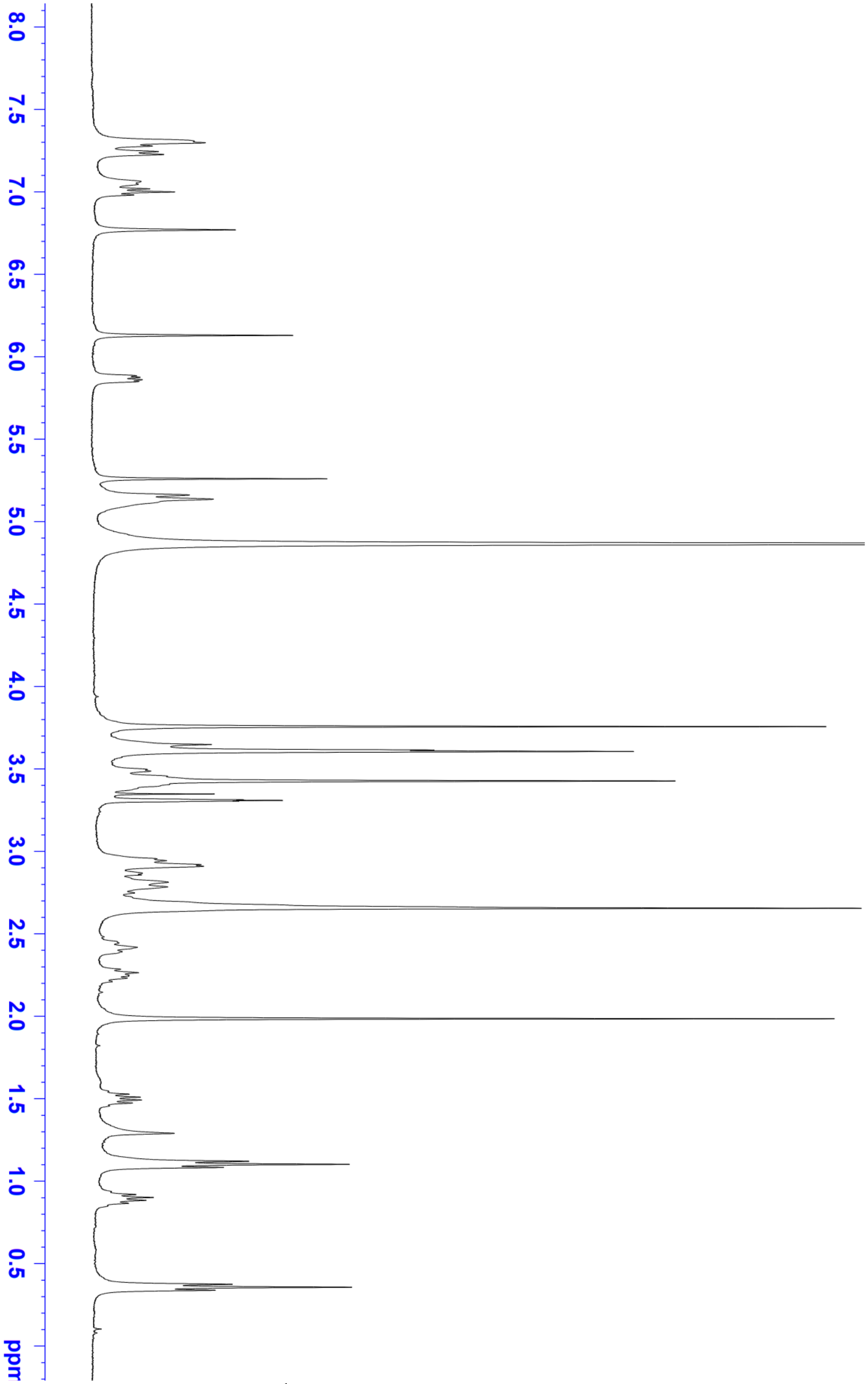

Figure S20. ^{1}H NMR spectrum of cathagine D (**4**) in CD_3OD.

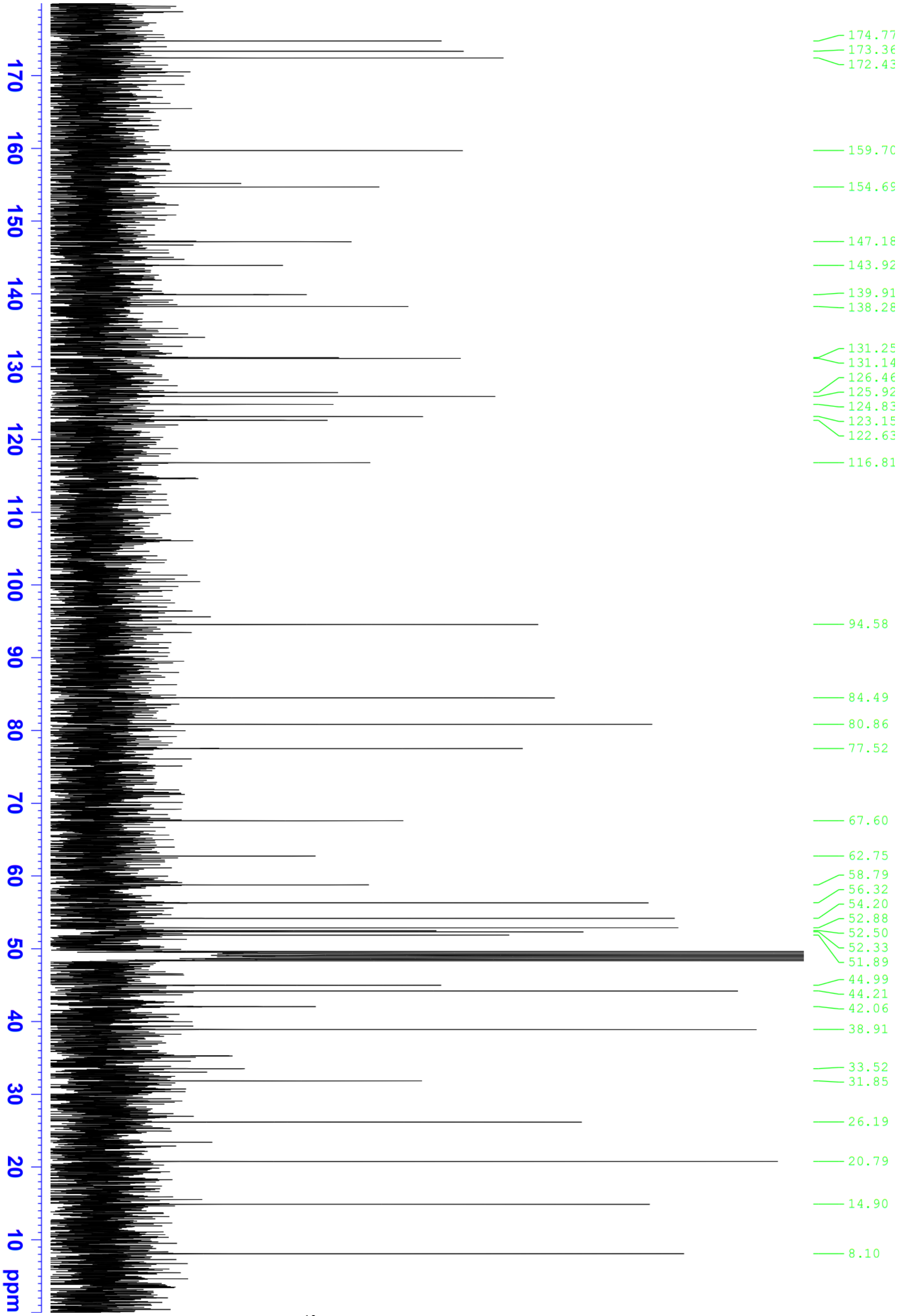

Figure S21. ^{13}C NMR spectrum of cathagine D (**4**) in CD_3OD.

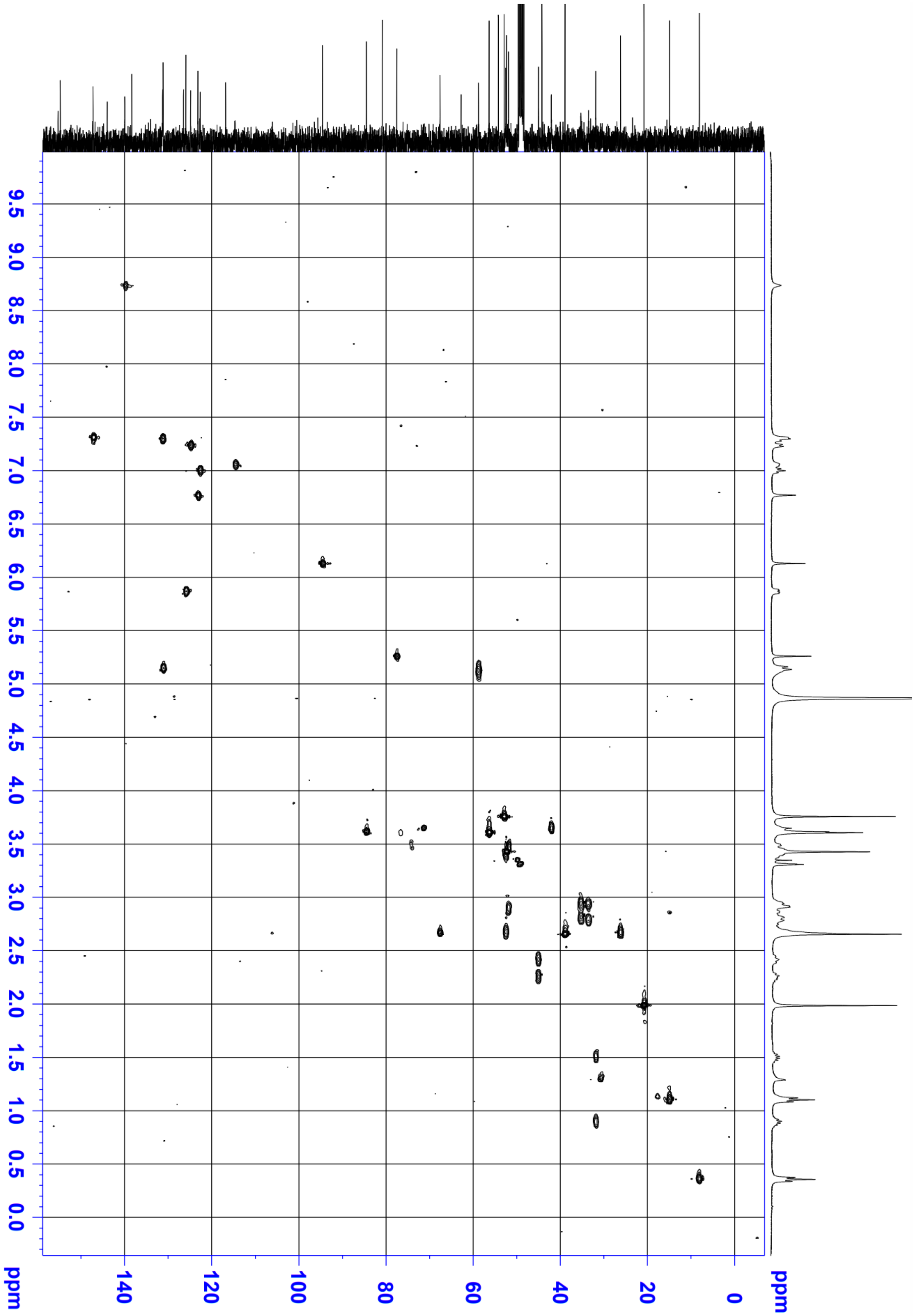

Figure S22. HSQC spectrum of cathagine D (**4**) in CD_3OD.

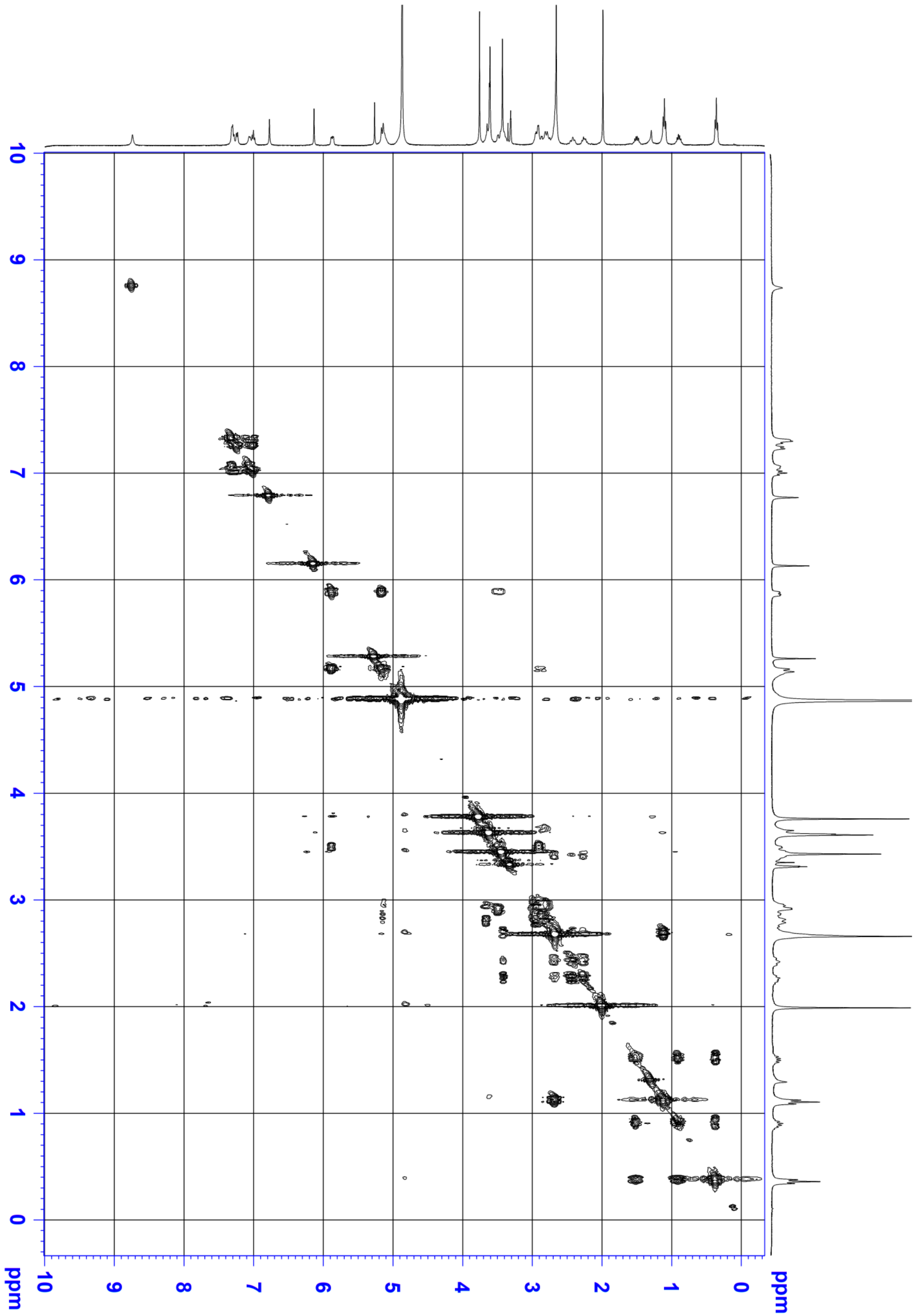

Figure S23. ^{1}H-^{1}H COSY spectrum of cathagine D (**4**) in CD_3OD.

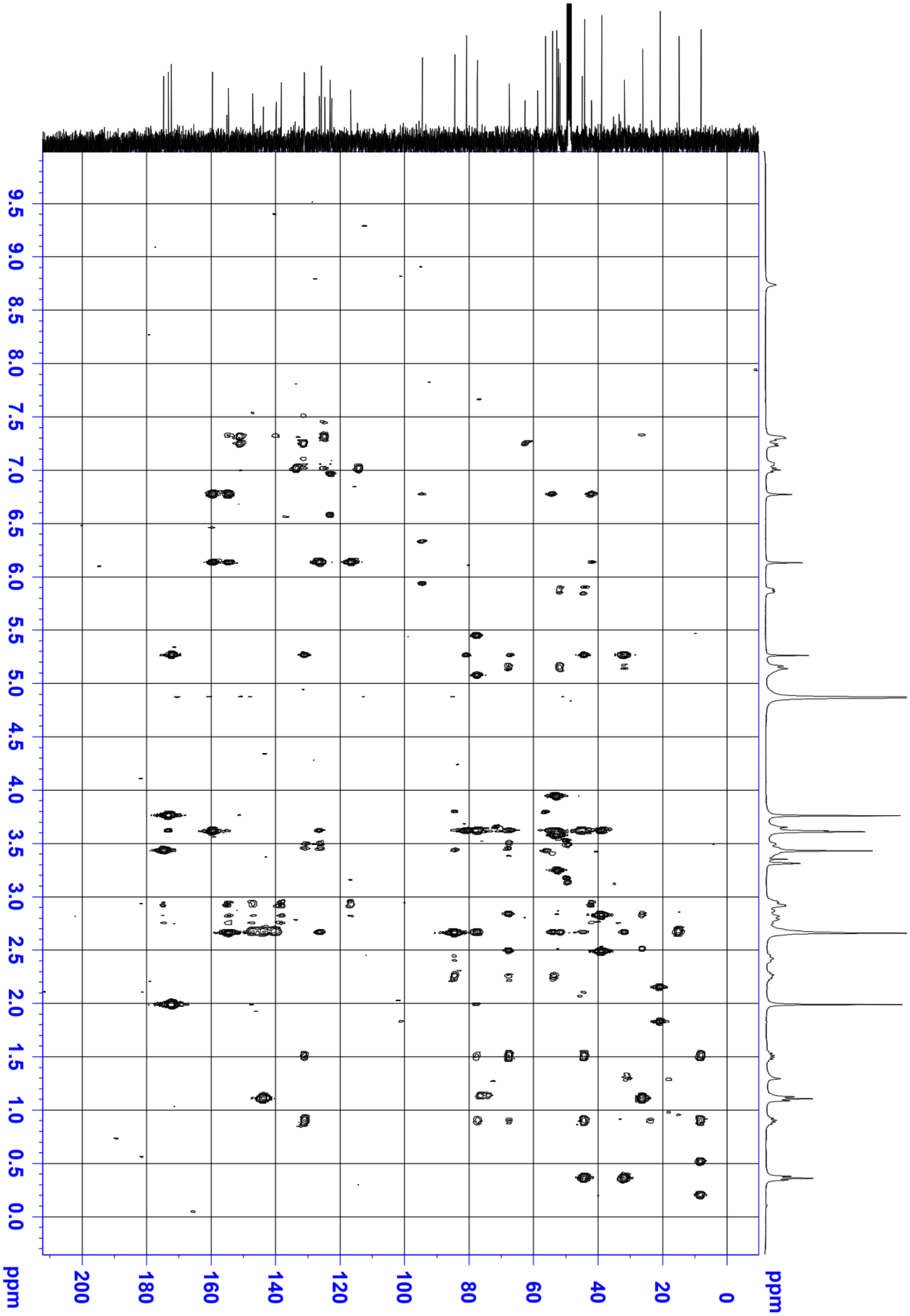

Figure S24. HMBC spectrum of cathagine D (**4**) in CD_3OD.

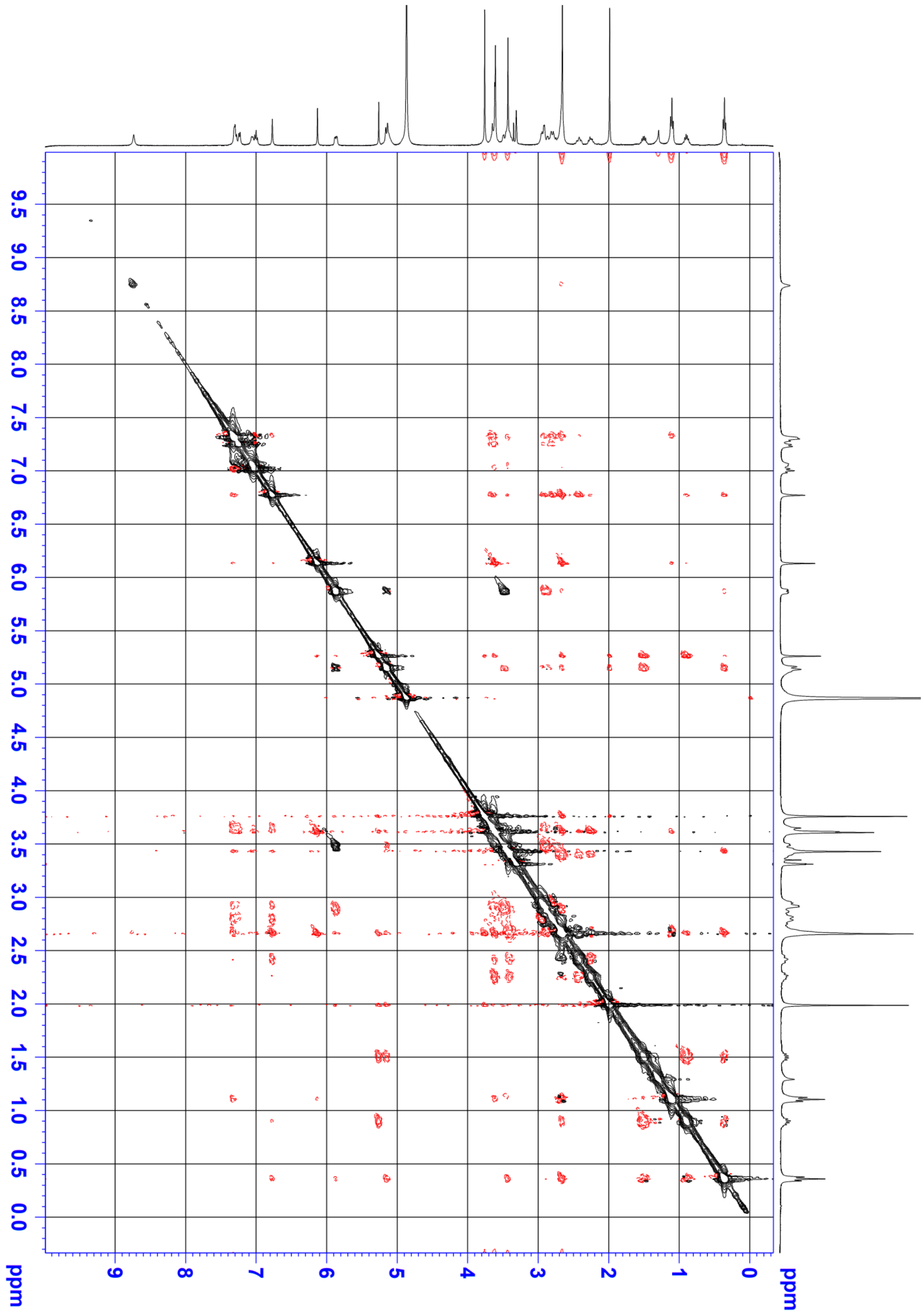

Figure S25. ROESY spectrum of cathagine D (**4**) in CD_3OD.
